# Cancer Subtype Discovery and Biomarker Identification via a New Robust Network Clustering Algorithm

**DOI:** 10.1371/journal.pone.0066256

**Published:** 2013-06-17

**Authors:** Meng-Yun Wu, Dao-Qing Dai, Xiao-Fei Zhang, Yuan Zhu

**Affiliations:** 1 Center for Computer Vision and Department of Mathematics, Sun Yat-Sen University, Guangzhou, China; 2 Department of Mathematics, Guangdong University of Business Studies, Guangzhou, China; New Jersey Institute of Technology, United States of America

## Abstract

In cancer biology, it is very important to understand the phenotypic changes of the patients and discover new cancer subtypes. Recently, microarray-based technologies have shed light on this problem based on gene expression profiles which may contain outliers due to either chemical or electrical reasons. These undiscovered subtypes may be heterogeneous with respect to underlying networks or pathways, and are related with only a few of interdependent biomarkers. This motivates a need for the robust gene expression-based methods capable of discovering such subtypes, elucidating the corresponding network structures and identifying cancer related biomarkers. This study proposes a penalized model-based Student’s t clustering with unconstrained covariance (PMT-UC) to discover cancer subtypes with cluster-specific networks, taking gene dependencies into account and having robustness against outliers. Meanwhile, biomarker identification and network reconstruction are achieved by imposing an adaptive 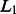 penalty on the means and the inverse scale matrices. The model is fitted via the expectation maximization algorithm utilizing the graphical lasso. Here, a network-based gene selection criterion that identifies biomarkers not as individual genes but as subnetworks is applied. This allows us to implicate low discriminative biomarkers which play a central role in the subnetwork by interconnecting many differentially expressed genes, or have cluster-specific underlying network structures. Experiment results on simulated datasets and one available cancer dataset attest to the effectiveness, robustness of PMT-UC in cancer subtype discovering. Moveover, PMT-UC has the ability to select cancer related biomarkers which have been verified in biochemical or biomedical research and learn the biological significant correlation among genes.

## Introduction

With the increasingly accumulation of genome-wide expression profiles, microarray-based method becomes a key technique for identifying cancer related genes (biomarkers) and discovering new cancer subtypes [1]. Compared with clinical and pathological risk factors, such as patient age, tumor size, and steroid receptor status, understanding the underlying genes can gain insight into cancer physiology [2]–[4], and is more effective for detection of new cancer subtypes, such as breast cancer [5], [6], ovarian cancer [7], colon cancer [8]. These subtypes may have differences in gene or protein expression, gene regulatory or protein signalling networks [9]. Predicting these subtypes from gene expression profiles can be viewed as a clustering problem, and finding the genes for prediction can be regarded as a problem of variable selection from high-dimensional unlabeled data.

One challenge of cancer subtype discovery is that the differences in network or pathway level across these subtypes may make the conventional clustering approaches based on gene expression profiles differences inadequate [9]. The discovery of these networks and pathways is very important in understanding the collective biological function of genes and their impact on the phenotypic changes of the patients [9]–[12]. In addition, biomarkers are often selected independently based on their discriminative abilities [13]. However, the genes often need to interact with others to participate in some biological processes or molecular functions [14]–[17]. Some of them may be not differentially expressed, but belong to a subnetwork which has overall discriminative activity or is a useful pathway for a specific subtype [3], [9], [18]. Therefore, the task of discovering the subtypes, elucidating their corresponding network structures, and picking out network-based biomarkers is still very important in biomedical fields.

There are various clustering methods applied on gene expression datasets for partitioning biological samples [19]. The model-based clustering which has a solid probabilistic framework is widely used in biomarker and cancer subtype discovering due to its good performance, interpretability and ease of implementation [20]. At present, the gene selection process of most approaches are designed by imposing penalty constraints on the likelihood to achieve a sparse solution.

For the penalized model-based clustering, in order to reduce the number of parameters, one common assumption is that each cluster has a diagonal covariance matrix, so the genes are assumed to be independent. Each cluster is often modeled as random variable drawn from mixture Gaussian distribution, and combined with several penalties, such as 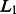 penalty, adaptive 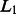 penalty and group 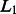 penalty [21], [22]. Since the log-probability of Gaussian distribution decays quadratically with distance from the center, it is sensitive to outliers which are commonly observed in microarray experiments due to either chemical or electrical reasons [23]. A more robust penalized model-based Student’s t clustering with diagonal covariance (PMT-DC) is introduced in [24] to deal with the noise and extreme genes. They also provide a way for ranking genes according to their contributions to the clustering process with a bootstrap procedure. However, the above methods ignore dependencies among genes within cancer subtypes. A regularized Gaussian mixture model is proposed to take various dependencies into account by permitting a treatment of general covariance matrices. An expectation maximization (EM) algorithm utilizing the graphical lasso is used for parameter estimation, and achieves better subtype discovering performance and gene selection [20]. As an intermediate between a diagonal and a general covariance matrix, another idea that modeling a covariance matrix using some latent variables as done in the mixture of factor analyzers is introduced [25]. It has more constrains and is more complex than the method based on an unconstrained covariance matrix. However, it is more effective if some latent variable-induced covariance assumption holds in the gene expression dataset. Both methods have difficult to deal with the outliers due to their Gaussian assumption. These conventional penalized model-based methods only select genes based on the mean response, and ignore their implications for the underlying networks or pathways which are very important in understanding the collective biological function.

Motivated by the challenges posed by the underlying networks or pathways and outliers observed in high-dimensional gene expression dataset, and the limitations of the above methods, this study proposes a penalized model-based Student’s t clustering with unconstrained covariance (PMT-UC) for cancer subtype discovery and biomarker identification. The new proposed method is based on multivariate Student’s t distribution which makes the algorithm not be affected by extreme or unusual genes. Unlike PMT-DC with the independent assumption, in order to consider the relationship between genes and discover the cancer subtypes which differ in terms of underlying network structures, a cluster-specific unconstrained covariance is used instead of diagonal covariance. The development of the algorithms for estimating sparse graphs by applying an 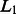 penalty to the inverse covariance matrix [26], [27] make the idea that taking gene dependence into account feasible. We impose an adaptive 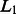 penalty on the means and the inverse scale matrices to achieve network-based biomarker identification and network reconstruction. The model is fitted via an EM algorithm by utilizing the graphical lasso. A new gene selection criterion is introduced to find the following informative genes: the genes that have cluster-specific means, the genes that are not differentially expressed but interact with some discriminative genes to form a collective biological function, and the genes which have class-specific underlying network structures. By applying the new model to simulated datasets and one publicly available cancer dataset, we show that the algorithm is robust against outliers on clustering, gene selection and network reconstruction processes simultaneously, and gives competitive results with the state-of-the-art algorithms on detecting new cancer subtypes. Many identified biomarkers have been verified in biochemical or biomedical research. The Gene Ontology (GO) analysis shows that the genes in the same subnetwork selected by the new proposed method have significant biological and functional correlation.

## Methods

This section introduces the penalized model-based Student’s t clustering with unconstrained covariance (PMT-UC) to select a few number of genes, that can be used to classify the 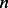 samples into naturally occurring groups, and to discover the relationship between the genes.

### The Framework of PMT-UC

Suppose that there are 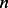 independent 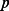-dimensional samples 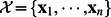, where 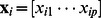 represents the gene expression of 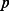 genes. The genes have been standardized to have a mean 0 and variance 1 across observations.

Each sample 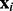 is supposed to come from a mixture distribution with 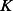 components of which the probability density function is
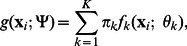
(1)where 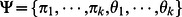 includes all the parameters in the model, 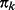 is the nonnegative mixing proportion for component 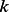 with 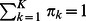, and 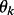 is the unknown parameters set corresponding to 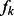.

Each component 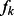 is specified as multivariate Student’s t distribution 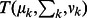 with the parameters set 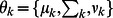, where 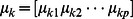 is the location parameter, 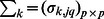 is the scale matrix and 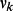 is the degrees of freedom. It has the probability density
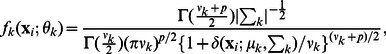
(2)where 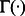 is the gamma function, and 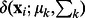 
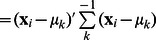
 denotes the Mahalanobis squared distance between 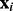 and 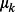. The mean and the covariance matrix of each Student’s t distribution is 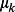 and 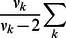, respectively. In general, the parameter set 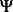 can be estimated by maximizing the log-likelihood function.

However, since the number of genes is often much more than the number of samples, the maximum likelihood estimation of 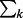 is probably singular. The inverse scale matrix 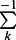 is denoted as 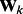 with the elements 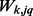. In the last few years, a number of authors introduce many approaches to yield a positive-definite covariance by increasing the sparsity of 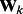 [26], [27]. The structure of a network is usually constructed based on correlation or partial correlation [28]. In this paper, the partial correlation 
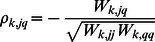
 can be derived from the inverse scale matrix. The partial correlation is used instead of correlation to present the relationship between two genes due to its ability of factoring out the influence of other genes. Therefore, 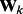 can reflect the relationship between the genes for cluster 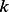 and can be regarded as the networks or pathways for genes. The statement that most genes (gene products) only interact with a few genes (gene products) indicates the sparsity of 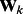 in terms of biological interpretation [15]. We impose an adaptive 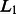 penalty on the off-diagonal elements of 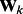 to deal with the sparsity of 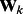 [29].

In addition, the sparsity of the mean 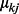 is considered, which is often used for gene selection. The mean-based discriminative gene is defined to have cluster-specific means, no matter whether it has a common or cluster-specific variances [20]. Specifically, it has at least one nonzero 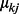 since the samples have been standardized to have mean 0 for each gene. Therefore, we impose an adaptive 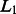 penalty on each 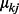 to shrink it to zero [29].

Then based on the penalized log-likelihood function which consists of log-likelihood function 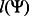 and penalty term 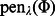, the objective function of PMT-UC to be maximized is as follows:
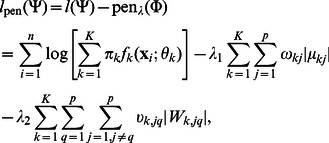
(3)where 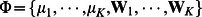, and 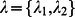 includes the non-negative regularization parameters 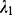 and 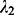 for 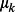
s and 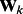
s respectively. The regularization parameters control the sparsity of the model. The larger the values of 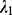 and 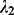, the more genes will be noninformative and independent. The adaptive 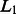 penalty is a weighted version of the 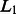 penalty with a weight 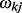 or 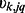 for each component. It achieves the three desirable properties simultaneously that can produce sparse solutions, ensure consistency of model selection, and result in unbiased estimates for large coefficients [30].

### Inference Algorithm

This study uses the expectation maximization (EM) algorithm [31] for optimizing the objective function 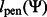 for 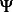 given fixed 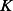 and 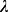. As in [20], [24], each sample 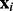 is assumed to have a corresponding unobserved indicator vector 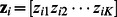, specifying the mixture component that 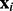 belongs to. If 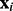 comes from component 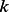 then 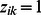, otherwise 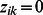. Given 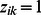, 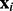 follows a Student’s t distribution with the probability density function 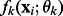. According to the fact that the Student’s t distribution can be written as a multivariate Gaussian distribution with the covariance matrix scaled by the reciprocal of a Gamma random variable, the additional missing data 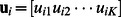 is introduced, where each element 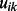 of 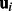 follows the Gamma distribution [32]. Then the penalized complete-data log-likelihood of the complete data 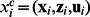 is
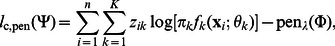
(4)where 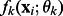 can be expressed as the product of the probability density functions of Gaussian and Gamma distributions (see Text S1 for details).

The EM algorithm iteratively applies an expectation (E) step to calculate the expected value 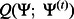 of 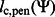 with respect to the current estimation 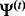 of the parameters at the 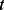
th iteration, and a maximization (M) step to find the updated parameters 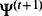 by maximizing 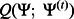, until achieving a stopping criterion 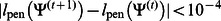.

**E step.** The value of 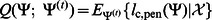 depends on the following three expectations (see Text S2 for details).

Since 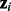 follows the Multinomial distribution and 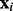 comes from the mixture distribution with probability density function 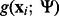, the value of 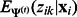 is given by
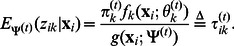
(5)



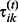
 can be regarded as the posterior probability of 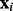 belonging to the 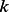
th cluster. Seeing that the Gamma distribution is conjugate to itself (self-conjugate) with respect to a Gaussian likelihood function, we have
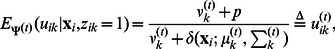
(6)and



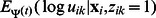


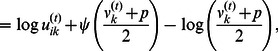
(7)where 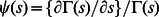 is the Digamma function [32].

**M step.** Firstly, the update of 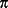 is given by the equation
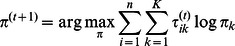
with the constraint 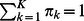 as



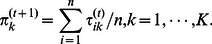
(8) Secondly, the value of 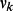 at the 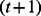
th iteration is a solution of the equation
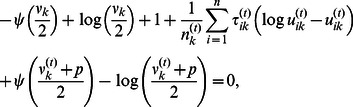
(9)where 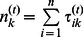. In this paper, since the solution of (9) is in non-closed form, the R function “nlminb” is used to find the numerical solution for 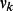 [24].

Thirdly, the aim is to maximize
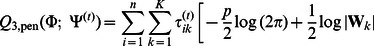


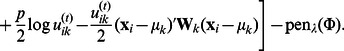
(10)to obtain the update for 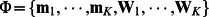. In the 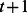 step, the adaptive weights are defined to be



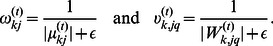
(11)The parameter 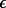 is introduced in order to provide stability and to ensure that a zero-valued component can escape from zero in the next iteration [33]. When 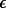 is too small, the zero-valued component still has so large weight that it will remain zero in the next iteration. When 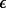 is too large, it makes the difference between the 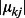
s or 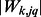
s not significant and allows many nonzero-valued components, resulting in a complex and inaccurate model. It has been assigned several values during the experiment procedure. It is shown that 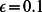 is appropriate. The initial estimates 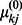 and 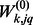 are chosen as the results estimated by the 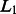 penalty.

By considering the differentiability of 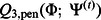 with respect to 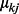 for two cases that 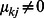 and 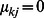, the updating estimate 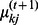 is as follows (see Text S3 for details) [20]: if
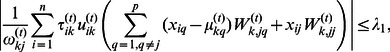
(12)then 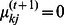; otherwise



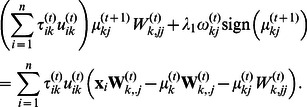
(13)After dropping the terms unrelated to 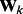 in 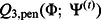, we have
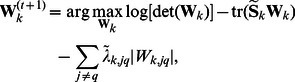
(14)where



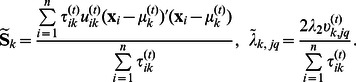



This optimization problem can be solved using the graphical lasso of which the corresponding R package “glasso” is available on CRAN [27]. The graphical lasso is designed to consider the problem of estimating sparse graphs by a lasso penalty applied to the inverse covariance matrix [27]. It is first proposed for the maximization of the Gaussian log-likelihood of the data with respect to the covariance matrix. The new proposed method takes 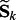 into account instead of the sample covariance matrix, where 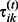 contains a posteriori information of the sample, and 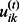 can reduce the effect of the outliers on this optimization problem.

### Model Selection

There are three parameters that need to be estimated before the PMT-UC algorithm, including the number of clusters 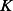, the penalization parameters 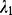 and 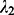. In this paper, the following approximate weight of evidence (AWE) criterion based on an approximation to the classification log-likelihood is used for model selection:
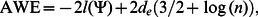
(15)where 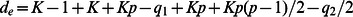 is the effective number of parameters in the model with 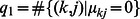 and 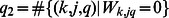 [34], [35]. It imposes a higher penalty on more complex model than BIC and is able to identify the correct number of clusters even when the component densities are misspecified [36], [37]. A grid search is applied to find the optimal 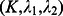 which has the minimum AWE.

### Subtype Discovering via Clustering

After the estimation of the parameters in PMT-UC, clusters can then be defined as samples following the similar distribution which is determined by the value of the posterior probability 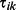. Given a sample, PMT-UC predicts the cancer subtype 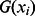 of the gene expression profile 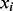 by that which gives the largest posterior probability 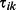, that is 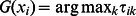.

### Elucidating the Underlying Network Structures

We can then elucidate the cluster-specific underlying network structures based on the inverse scale matrix 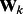. A cluster-specific network can be represented as undirected graph, with the genes as the vertices and edges as their relationships based on 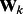. Edges connect those genes whose partial correlations derived from 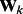 are larger than 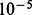. Then a subnetwork is defined as a set containing genes and edges that induces a single connected component in this network. These cluster-specific subnetworks indicate the different relationships among genes with various cancer subtypes and are regarded as the underlying network structures.

### Network-based Biomarker Identification

Due to that the genes in a cell seldom act alone, but form a network of interactions [14], the biomarkers are identified as subnetworks of interacting genes instead of individual genes in this paper. Specifically, we firstly pick out the subnetworks defined above. Secondly, in consideration of the fact that the noisy gene and the informative gene are uncorrelated with each other [20], [38], the subnetworks that have at least one mean-based discriminative gene are chosen as subnetwork biomarkers. This gene selection criterion can identify genes that are not differentially expressed but interact with some discriminative genes to form a collective biological function. Finally, the remaining subnetworks of which the internal structure (the relationship between the genes) are different among 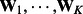 are also regarded as biomarkers to elucidate the cluster-specific underlying network structures.

### The Final Algorithm for PMT-UC

**Figure 1:**
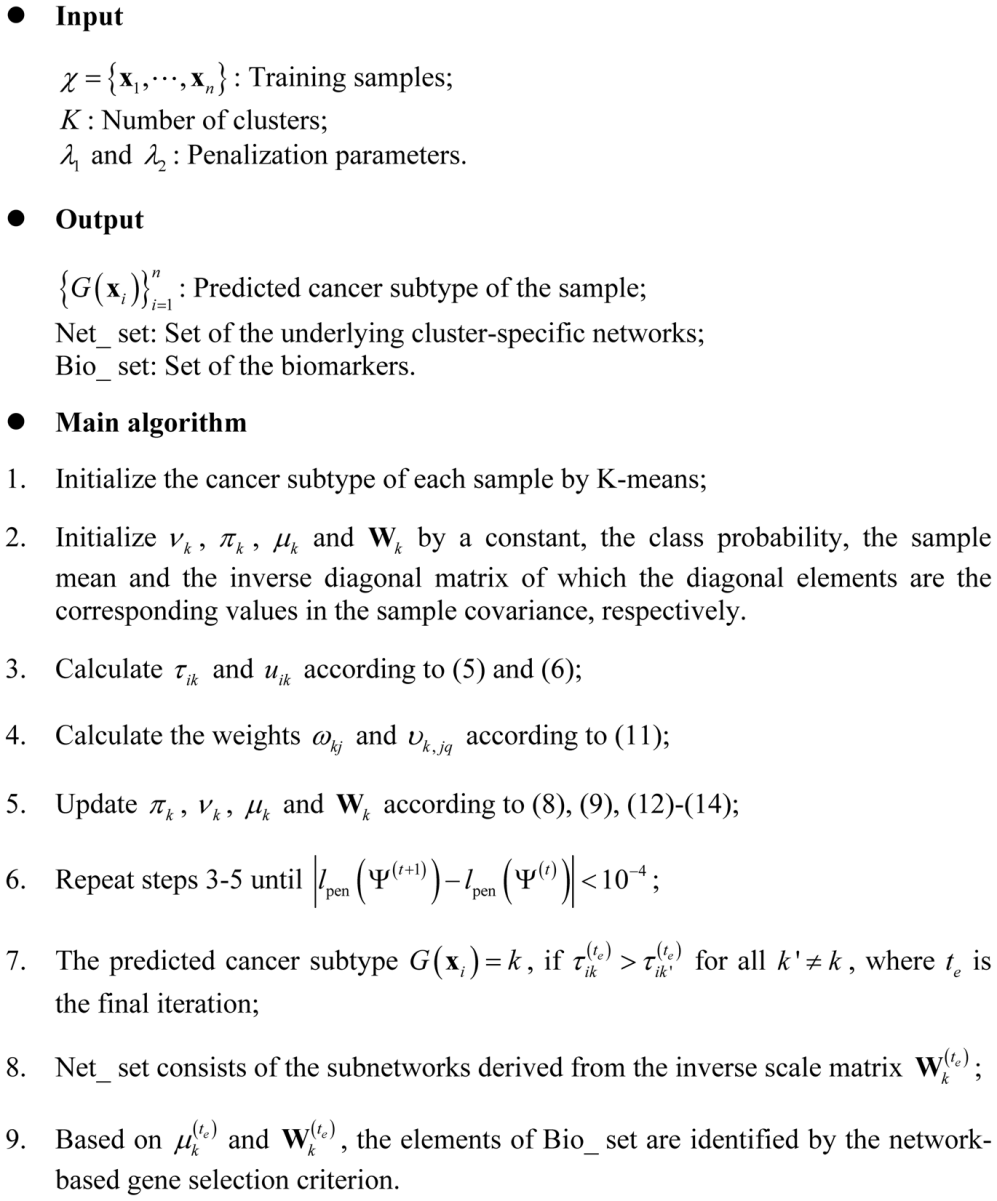
Summary of PMT-UC for discovering cancer subtypes, underlying network structures, and biomarkers.

Figure 1 summarizes the detailed algorithm for discovering cancer subtypes, underlying network structures, and network-based biomarkers via PMT-UC. For any given 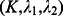, the result of K-means is used as the initialization for the EM algorithm. In order to avoid the local optimum of K-means, we run the entire algorithm five times with random K-means initialization, and choose the result that gives the highest value of objective function (3).

## Results and Discussion

### Simulations

A dataset with redundant genes is simulated to evaluate the clustering, gene selection and network reconstruction performance of the method. The dataset has 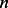 samples and 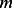 informative genes with input dimension 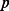. 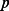 is taken to be higher than sample size of each cluster so that the sample covariance of each cluster is not reversible. The first 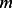 informative genes come from a 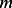-dimensional multivariate Student’s t distribution 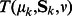 for the 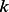
th cluster. The remaining 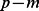 noisy genes which are independent of the informative genes are independently and identically distributed from univariate Student’s t distribution 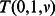 for all clusters. The degrees of freedom 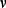 will affect the noise level of the dataset. The lower the degrees of freedom the fatter tails the dataset will have.

Firstly, the dataset with two clusters is simulated, having 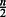 samples for each cluster. Three cases 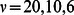 are considered in the next experiments to explore the effects of the outliers on the performance of the method [24]. When 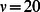, the distribution of the simulated dataset is approximate to Gaussian distribution. For each of the three cases, the following four set-ups are considered:

- set-up 1 has cluster-specific means with 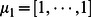 and 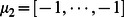, and common diagonal scale matrix with 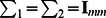, where 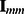 is a 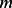-dimensional identity matrix.
- set-up 2 has cluster-specific means with 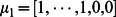 and 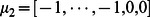, and common non-diagonal scale matrix with 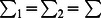. 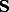 is a sparse symmetry matrix that has the diagonal elements 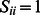 and the non-diagonal elements 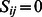 with the exception of 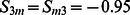, 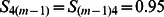.
- set-up 3 has cluster-specific means with 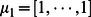 and 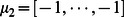, and uses two general sparse scale matrices generated by the similar procedure described in [9], [26]. A diagonal matrix with same positive diagonal entries is generated firstly, then a given number of nonzeros are randomly inserted in the non-diagonal locations of specified section 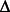 of the matrix symmetrically. The number of nonzero non-diagonal entries is set to 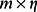. A multiple of the identity is adding to the matrix to ensure the positive definiteness. Finally, each element is divided by the corresponding diagonal element to generate the inverse scale matrix. In this set-up, 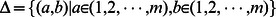 and 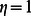.
- set-up 4 has cluster-specific means with 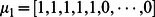 and 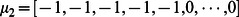, and similar non-diagonal scale matrices as set-up 3 with 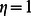 and 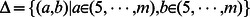.

Under the simulated pattern stated above, we set 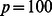, 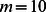 and 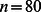 similar to that introduced in [20]. For each set-up, the simulation is repeated 50 times and fitted with 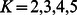, 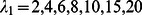, and 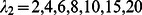.

PMT-UC is compared with penalized model-based Gaussian clustering with unconstrained covariance (PMG-UC) and penalized model-based Student’s t clustering with diagonal covariance (PMT-DC) in terms of the following evaluation criterions. The Rand Index (RI), the adjusted Rand Index (aRI) and the frequencies of the selected numbers (N) of clusters (K) are used to assess the ability of the method for clustering [20]. In order to quantify the ability of the method for network reconstruction, the structural hamming distance (SHD) between true and inferred networks is computed, which is the number of edge differences to transform one network to another network [9]. The smaller SHD indicates the closer approximation to the true network. The following two indexes are used for evaluation of the gene selection performance, the number of informative variables incorrectly selected to be noninformative (false negatives, FN) and the number of noninformative variables correctly selected (true negatives, TN) [20].

#### Effect of the parameter 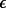


The effect of the parameter 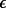 which is designed for the stability of the algorithm on the performance of PMT-UC is discussed in terms of the five measures introduced above (RI, aRI, SHD, FN and TN). Particularly, we run PMT-UC on a fixed dataset under the set-up 4 with 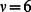 of which the dataset has higher noise level, a fewer genes with cluster-specific means and some genes with cluster-specific network structures, with different values of 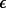 (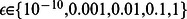). Table 1 shows the averages and standard deviations of five measures in 50 simulations with respect to various values of 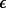 on this set-up. When 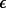 is not too large, the algorithm performance tends to be fairly robust to the choice of 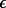. Since the results with 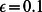 show some improvement over the other situations, 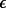 is set to 0.1 in the following experiments.

**Table 1:** The effect of the parameter 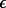 on the performance of PMT-UC.

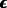	RI	aRI	SHD1	SHD2	FN	TN
10^–10^	0.918 (0.045)§	0.836 (0.090)	5.000 (0.798)	4.565 (1.727)	2.870 (1.792)	89.609 (0.583)
0.001	0.923 (0.048)	0.846 (0.095)	4.913 (0.949)	4.826 (1.072)	2.174 (1.557)	89.565 (0.590)
0.01	0.914 (0.049)	0.828 (0.098)	5.435 (1.472)	5.043 (1.107)	2.609 (2.210)	89.174 (1.029)
0.1	0.937 (0.034)	0.873 (0.068)	2.652 (1.229)	2.522 (1.344)	0.870 (0.968)	90.000 (0.000)
1	0.689 (0.192)	0.380 (0.383)	5.000 (0.000)	5.261 (0.864)	6.913 (2.575)	88.478 (1.702)

The effect of the parameter 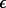 on the performance of PMT-UC is discussed in terms of the five measures RI, aRI, SHD, FN and TN, where SHD1 and SHD2 are the results for the first and second clusters respectively, FN is the number of informative variables incorrectly selected to be noninformative and TN is the number of noninformative variables correctly selected. In the true case, 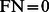, 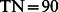.

§ c(d): c and d are the average and standard deviation of corresponding results in 50 simulations, respectively.

#### Effect of the initialization

The convergence of PMT-UC is studied by considering the corresponding results with respect to different initializations using K-means. This study also depends on the set-up 4 with 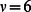. A simulated dataset is fixed and the entire procedure is applied ten times of which each time uses five K-means initializations. The standard deviations of the selected parameters and experiment results of these ten experiments can be regarded as the evaluation indexes for the convergence of PMT-UC. To reduce the variability, five datasets are generated, and the averages and standard deviations of results for each dataset are list in Table 2. It is shown that the clustering and gene selection results do not have significant change with different initializations. However, the complete PMT-UC algorithm has a certain variance in terms of the parameter 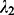 and the results SHD that correspond to network reconstruction.

**Table 2:** The convergence of PMT-UC with respect to different initializations.

dataset	1	2	RI	aRI	SHD1	SHD2	FN	TN
1	4.0 (0.0)§	3.6 (0.5)	0.91 (0.04)	0.83 (0.07)	3.00 (1.58)	2.00 (1.73)	0.40 (0.89)	90.00 (0.00)
2	4.0 (0.0)	4.0 (0.0)	0.97 (0.01)	0.94 (0.02)	3.20 (1.48)	3.00 (1.58)	0.80 (0.45)	90.00 (0.00)
3	4.0 (0.0)	3.8 (0.4)	0.95 (0.01)	0.89 (0.02)	1.80 (0.45)	3.60 (0.55)	1.20 (0.45)	90.00 (0.00)
4	4.0 (0.0)	4.0 (0.0)	0.95 (0.02)	0.89 (0.04)	6.40 (1.34)	4.00 (0.71)	1.60 (0.89)	89.80 (0.45)
5	4.0 (0.0)	4.0 (0.0)	0.95 (0.00)	0.90 (0.00)	1.40 (0.55)	3.60 (0.55)	1.00 (0.00)	90.00 (0.00)

The convergence of PMT-UC is explored by considering the selected parameters 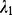 and 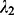, and the experiment results RI, aRI, SHD, FN and TN, with respect to different initializations using K-means. SHD1 and SHD2 are the results for the first and second clusters respectively, FN is the number of informative variables incorrectly selected to be noninformative and TN is the number of noninformative variables correctly selected. In the true case, 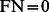, 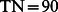.

§ c(d): c and d are the average and standard deviation of corresponding results in 10 experiments with a fixed dataset, respectively.

#### Clustering results

The experiment clustering results of the four set-ups with 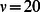 are shown in Table 3. Since the datasets come from an approximate distribution of Gaussian distribution, both PMT-UC and PMG-UC always correctly identify the two clusters. For set-ups 1, 2, 3, PMT-UC works slightly better than PMG-UC in identifying clustering structures, as summarized by the RI or aRI in Table 3. For set-up 4, with the presence of more noise variables based on the mean, RI and aRI of PMG-UC decrease dramatically to 0.734 and 0.47. For set-up 1 with the true model with a diagonal covariance matrix, both PMT-UC and PMT-DC have similar clustering performances. The stronger the correlations among variables, the more likely for the PMT-DC to get more clusters by mistake and have poor clustering performance. Especially, for PMT-DC with the independence assumption, the dataset in set-up 4 only has five informative genes, which results in high clustering error rate.

**Table 3:** Comparison of performance of PMT-UC, PMG-UC and PMT-DC applied on binary-clusters simulated datasets.

ν	Set-up	*Κ*	PMT-UC					PMG-UC					PMT-DC				
			N	RI	aRI	FN	TN	N	RI	aRI	FN	TN	N	RI	aRI	FN	TN
20	1	2	**50**	**0.994**	**0.988**	**0.00**	**90.00**	**50**	**0.983**	**0.975**	**0.00**	84.36	45	**0.990**	**0.979**	**0.00**	**89.09**
		3	0	–	–	–	–	0	–	–	–	–	5	**0.978**	0.956	**0.00**	86.20
		4/5‡	0	–	–	–	–	0	–	–	–	–	0	–	–	–	–
		A†	50	**0.994**	**0.988**	**0.00**	**90.00**	50	**0.983**	**0.975**	**0.00**	84.36	50	**0.988**	**0.977**	**0.00**	**88.80**
	2	2	**50**	**0.998**	**0.995**	**0.00**	**89.80**	50	0.971	0.941	1.86	84.42	40	**0.981**	0.962	2.00	**89.47**
		3	0	–	–	–	–	0	–	–	–	–	10	0.960	0.919	2.00	84.80
		4/5	0	–	–	–	–	0	–	–	–	–	0	–	–	–	–
		A	50	**0.998**	**0.995**	**0.00**	**89.80**	50	0.971	0.941	1.86	84.42	50	**0.977**	0.953	2.00	**88.54**
	3	2	**50**	**0.995**	**0.990**	**0.00**	**89.80**	**50**	**0.987**	**0.975**	**0.00**	84.72	23	**0.995**	**0.989**	**0.00**	**89.35**
		3	0	–	–	–	–	0	–	–	–	–	22	0.929	0.857	**0.00**	85.59
		4/5	0	–	–	–	–	0	–	–	–	–	5	0.861	0.721	**0.00**	76.60
		A	50	**0.995**	**0.990**	**0.00**	**89.80**	50	**0.987**	**0.975**	**0.00**	84.72	50	0.952	0.904	**0.00**	86.42
	4	2	**50**	**0.944**	**0.889**	**0.00**	**90.00**	**50**	0.734	0.470	7.22	**88.84**	39	0.883	0.767	5.51	**89.64**
		3	0	–	–	–	–	0	–	–	–	–	11	0.841	0.681	5.00	87.64
		4/5	0	–	–	–	–	0	–	–	–	–	0	–	–	–	–
		A	50	**0.944**	**0.889**	**0.00**	**90.00**	50	0.734	0.470	7.22	**88.84**	50	0.874	0.748	5.40	**89.20**
10	1	2	**50**	**0.983**	**0.966**	**0.00**	**89.92**	41	0.942	0.884	**0.78**	83.24	28	**0.976**	**0.952**	**0.36**	**88.89**
		3	0	–	–	–	–	9	0.881	0.761	**0.00**	76.33	19	0.943	0.885	**0.00**	79.21
		4/5	0	–	–	–	–	0	–	–	–	–	3	0.891	0.782	**0.00**	87.33
		A	50	**0.983**	**0.966**	**0.00**	**89.92**	50	0.931	0.862	**0.64**	82.00	50	0.958	0.917	**0.20**	85.12
	2	2	**50**	**0.988**	**0.976**	**0.00**	**89.92**	46	0.867	0.734	2.70	82.13	36	0.943	0.887	2.28	**89.11**
		3	0	–	–	–	–	4	0.797	0.593	1.50	72.50	13	0.943	0.887	1.92	80.31
		4/5	0	–	–	–	–	0	–	–	–	–	1	0.882	0.764	4.00	85.00
		A	50	**0.988**	**0.976**	**0.00**	**89.92**	50	0.861	0.723	2.60	81.36	50	0.942	0.885	2.22	86.74
	3	2	**50**	**0.992**	**0.984**	**0.00**	**88.88**	33	0.922	0.845	**0.00**	82.52	16	0.873	0.747	1.63	**88.19**
		3	0	–	–	–	–	17	0.853	0.706	**0.00**	55.53	28	0.942	0.884	**0.00**	82.96
		4/5	0	–	–	–	–	0	–	–	–	–	6	0.758	0.516	1.67	80.50
		A	50	**0.992**	**0.984**	**0.00**	**88.88**	50	0.899	0.798	**0.00**	73.34	50	0.898	0.796	**0.72**	84.34
	4	2	**50**	**0.949**	**0.899**	**0.00**	**89.50**	**50**	0.499	0.000	8.80	76.70	42	0.681	0.368	7.02	86.64
		3	0	–	–	–	–	0	–	–	–	–	5	0.796	0.593	4.80	85.40
		4/5	0	–	–	–	–	0	–	–	–	–	3	0.644	0.284	8.33	84.33
		A	50	**0.949**	**0.899**	**0.00**	**89.50**	50	0.499	0.000	8.80	76.70	50	0.691	0.385	6.88	86.38
6	1	2	50	**0.961**	**0.922**	**0.00**	**89.70**	40	0.619	0.240	6.88	74.75	32	**0.957**	**0.914**	**0.63**	**89.75**
		3	0	–	–	–	–	10	0.868	0.735	0.00	81.50	13	0.872	0.743	0.92	84.77
		4/5	0	–	–	–	–	0	–	–	–	–	5	0.503	0.008	4.00	90.00
		A	50	**0.961**	**0.922**	**0.00**	**89.70**	50	0.669	0.339	5.50	76.10	50	0.889	0.779	**1.04**	**88.48**
	2	2	**50**	**0.980**	**0.961**	**0.00**	**90.00**	45	0.550	0.101	8.82	78.20	46	0.672	0.351	4.59	83.00
		3	0	–	–	–	–	5	0.863	0.726	2.00	75.00	0	–	–	–	–
		4/5	0	–	–	–	–	0	–	–	–	–	4	0.501	0.001	10.00	88.50
		A	50	**0.980**	**0.961**	**0.00**	**90.00**	50	0.581	0.163	8.14	77.88	50	0.659	0.323	5.02	83.44
	3	2	**50**	**0.952**	**0.904**	**0.10**	**89.80**	45	0.502	0.006	9.89	**88.33**	27	0.542	0.094	7.85	80.93
		3	0	–	–	–	–	5	0.500	0.004	9.00	82.00	15	0.749	0.500	2.00	73.07
		4/5	0	–	–	–	–	0	–	–	–	–	8	0.617	0.232	7.50	78.75
		A	50	**0.952**	**0.904**	**0.10**	**89.80**	50	0.502	0.006	9.80	87.70	50	0.616	0.238	6.04	78.22
	4	2	**50**	**0.935**	**0.870**	**0.40**	**90.00**	**50**	0.498	0.002	9.90	87.10	39	0.495	0.000	8.10	76.44
		3	0	–	–	–	–	0	–	–	–	–	7	0.507	0.019	5.86	67.71
		4/5	0	–	–	–	–	0	–	–	–	–	4	0.619	0.234	1.00	67.00
		A	50	**0.935**	**0.870**	**0.40**	**90.00**	50	0.498	0.002	9.90	87.10	50	0.506	0.021	7.22	74.46

The clustering and gene selection results for the four set-ups with 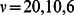, in terms of the average frequencies (N) of the selected numbers of clusters (K), and the average of RI, aRI, FN, and TN in 50 simulations, where FN is the number of informative variables incorrectly selected to be noninformative and TN is the number of noninformative variables correctly selected. In the true case, 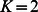, 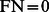, 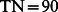. The table indicates in bold all results that perform best or that are not significantly different from each other.

‡ : 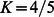 denotes that 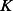 can be 4 or 5.

† : 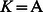 denotes that 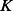 can be any element of the set 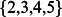 which contains the predefined numbers of clusters.

To investigate the effect of the outliers, we use the smaller degrees 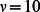 and 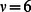. Table 3 also gives the results for the four set-ups with these two cases. As expected, PMG-UC performs poorly with smaller degrees, and it is more sensitive to extreme observations. For set-up 1, the clustering results of PMT-DC do not change significantly with the decreasing of degrees for its robustness and independence assumption. However, it often can not find the true clustering structures in the other three set-ups. In summary, the results for set-ups 1–4 when 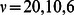 demonstrate that PMT-UC has better clustering performance than PMG-UC and PMT-DC for the datasets with independent or correlated informative genes, and is robust to the outliers.

#### Network reconstruction

**Figure 2:**
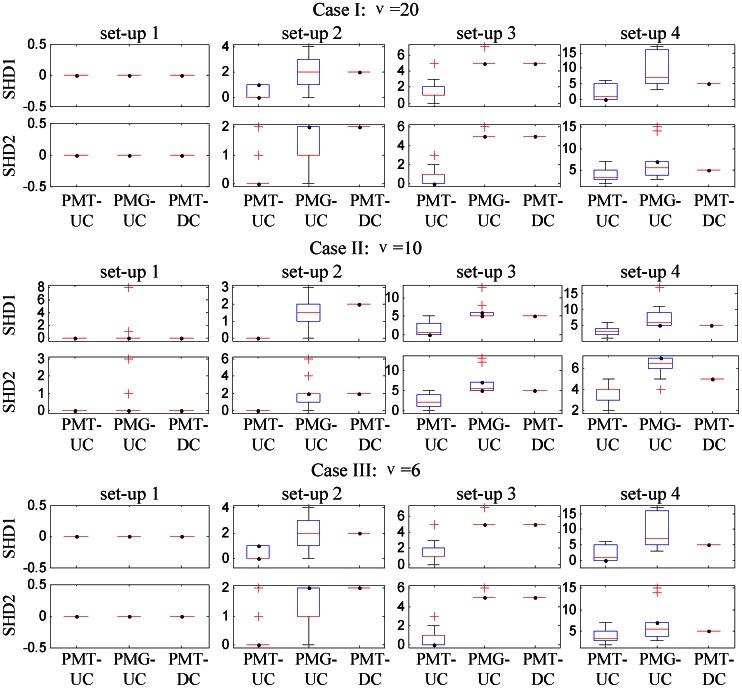
Boxplots of structural hamming distance (SHD) between correct and inferred networks. On each box, the central mark is the median, the edges of the box are the 25th and 75th percentiles, the whiskers extend to the most extreme data points not considered outliers, and outliers are plotted individually. Results shown for PMT-UC, PMG-UC and PMT-DC in the four set-ups of three cases 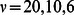. SHD1 and SHD2 are the results for the first and second clusters, respectively.

**Figure 3:**
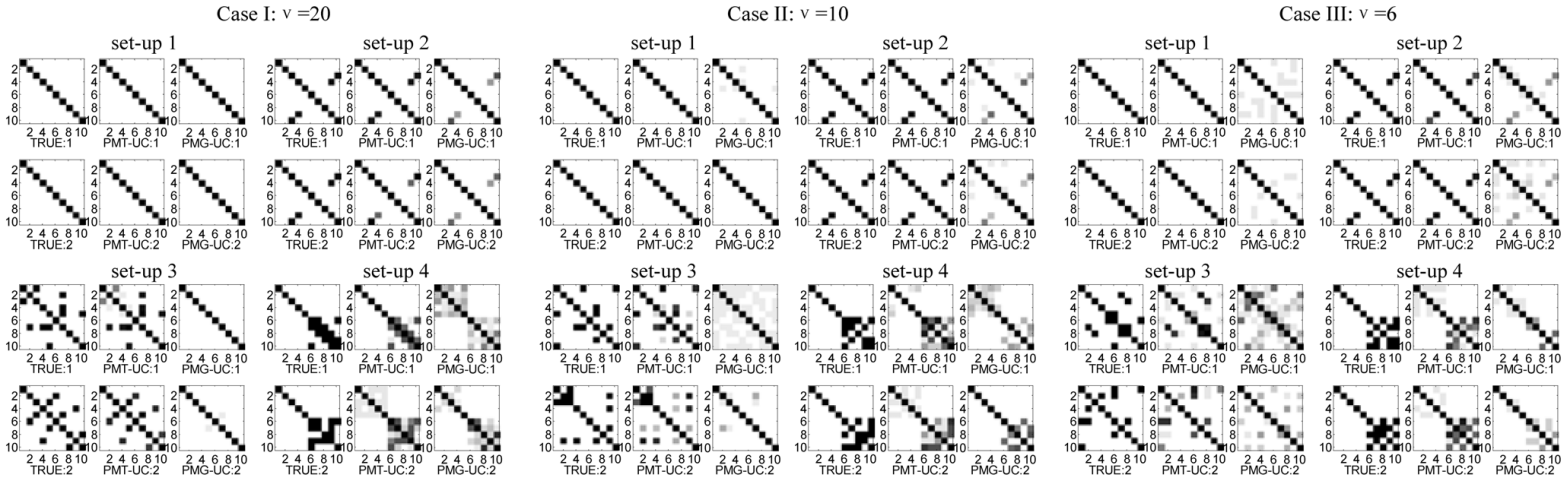
Network reconstruction for simulated datasets with 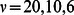. TRUE:1 and TRUE:2 are the parts of the original 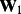 and 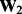 corresponding to the first 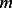 informative genes for the first and second clusters, respectively. PMT-UC:1 and PMT-UC:2 are the estimation of those parts of the inverse scale matrices using PMT-UC. PMG-UC:1 and PMG-UC:2 are the estimation of those parts of the inverse covariance matrices using PMG-UC.

Figure 2 shows the boxplots of cluster-specific SHD between estimated and true networks over 50 simulations for the above four set-ups of the three cases when 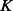 is set to 2. In addition, we plot the average sparsity pattern which is the relative frequency matrix for PMG-UC and PMT-UC. Since PMT-DC assumes a diagonal covariance, it is not plotted here. The relative frequency matrix is comprised of the relative frequency of nonzero estimated of each element of the inverse scale matrix 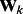 over the 50 repetitions. Figure 3 shows the cluster-specific results of the first 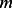 informative genes (see Text S4 for the results of the total 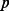 genes). We make the following observations based on the results given in Figures 2 and 3. At all the cases, PMT-UC provides smallest SHD relative to the other two approaches. When 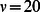 with which the Student’s t distribution is similar to Gaussian distribution, both PMT-UC and PMG-UC are able to recover the sparse inverse covariance structure for set-up 1. It is shown that although both PMT-UC and PMG-UC have non-diagonal assumption, they can get the diagonal covariance as the truth by a sufficiently large penalty on the off-diagonal elements of the inverse covariance matrices. For set-up 2, PMT-UC can accurately identify the location of the nonzeros almost every simulation. Meanwhile, with the high value of the off-diagonal nonzeros of covariance, PMG-UC can also recover the inverse covariance pattern sometimes. However, when the partial correlations of the genes are not high in the set-up 3, with the 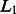 penalty, PMG-UC does not have good network reconstruction performance different from that of PMT-UC. For the set-up 4, with the increasing of the noise in terms of the mean, the result of PMG-UC is obscure. When 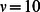 or with which the dataset has higher noise level, PMG-UC is unable to recover network structure. However, PMT-UC can still discover the relationship between genes under the network.

#### Gene selection

The two gene selection evaluation indexes FN and TN are also summarized in Table 3. For the four set-ups, PMG-UC tends to picks out more genes which are uninformative than PMT-UC and PMT-DC. In set-ups 1 and 3, the informative genes have cluster-specific means and can be selected by all the three methods when the dataset has low noise level. For set-ups 2 and 3, there are two genes which are not differentially expressed but interact with some discriminative genes, and five genes which are also not differentially expressed but have different underlying network structures, respectively. Table 3 shows that among the three methods only PMT-UC can discover these genes.

#### The dataset with multiple thin-tailed clusters

For 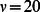, an additional dataset with more thin-tailed clusters is taken account into, where the number of clusters is assumed to be 5. The first two clusters are generated using the simulated pattern of set-up 4, where the values of relevant settings are not changed. The other three clusters contain more mean-based discriminative genes with 15 samples for each cluster, having 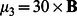, 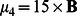, and 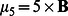, where 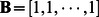, and common diagonal scale matrix. The model is fitted with 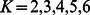, 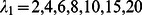, and 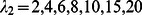. Table 4 presents the average results of the three algorithms in 50 simulations, including the RI and aRI with respect to the first two clusters and the other three ones. When the dataset has many thin-tailed clusters, PMT-UC tries to explain the first two clusters whose means are not too different by fat tails. Therefore, unlike the good clustering performance when the dataset has only two clusters, PMT-UC can not identify the true clustering structures of these two clusters although the informative genes are selected correctly. Since the model selection criterion of PMT-UC tends to select the sampler model with less nonzero parameters, it can not pick out the model with four or five clusters as PMT-DC does. Due to the bad initializations using K-means, PMG-UC also regards these two clusters as one although it is not so flexible as PMT-UC. The superiority of PMT-UC can not be reflected in the simulation having many thin-tailed clusters of which some clusters do not have enough mean-based discriminative genes. The good performance of algorithm may need more genes having cluster-specific means with the increasing of the number of clusters.

**Table 4:** Comparison of performance of PMT-UC, PMG-UC and PMT-DC applied on simulated datasets with multiple thin-tailed clusters.

Method	*Κ*	N	FN	TN	RI	aRI	RI_1_	aRI_1_	RI_2_	aRI_2_
PMT-UC	2	–	–	–	–	–	–	–	–	–
	3	50	0.00	90.00	0.639	0.347	1.000	1.000	0.494	0.000
	4	–	–	–	–	–	–	–	–	–
	5	–	–	–	–	–	–	–	–	–
	6	–	–	–	–	–	–	–	–	–
	A†	50	0.00	90.00	0.639	0.347	1.000	1.000	0.494	0.000
PMG-UC	2	–	–	–	–	–	–	–	–	–
	3	–	–	–	–	–	–	–	–	–
	4	8	0.00	89.00	0.794	0.445	1.000	1.000	0.495	0.000
	5	42	0.00	89.62	0.793	0.489	0.993	0.984	0.497	0.000
	6	–	–	–	–	–	–	–	–	–
	A	50	0.00	89.52	0.793	0.482	0.995	0.987	0.497	0.000
PMT-DC	2	–	–	–	–	–	–	–	–	–
	3	–	–	–	–	–	–	–	–	–
	4	39	0.00	88.77	0.794	0.564	1.000	1.000	0.496	0.000
	5	11	0.00	88.00	0.797	0.506	1.000	1.000	0.502	0.000
	6	–	–	–	–	–	–	–	–	–
	A	50	0.00	88.60	0.794	0.551	1.000	1.000	0.495	0.000

The comparison of performance of PMT-UC, PMG-UC and PMT-DC applied on simulated datasets with multiple thin-tailed clusters, in terms of the average frequencies (N) of the selected numbers of clusters (K), and the average of RI, aRI, FN, and TN in 50 simulations. RI_1_ and RI_2_ are the RI with respect to the first two clusters and the last three clusters, respectively. aRI_1_ and aRI_2_ are the aRI with respect to the first two clusters and the last three clusters, respectively. In the true case, 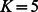, 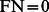, 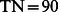.

† : 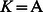 denotes that 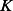 can be any element of the set 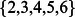 which contains the predefined numbers of clusters.

### Application to Real Dataset

In order to evaluate clustering capability, gene selection and network reconstruction performance of PMT-UC, experiments are carried out on one publicly available cancer dataset. This dataset is the expression profiles of 7129 genes on 72 acute leukemia samples described by Golub *et al.* [39]. It includes 47 samples of acute lymphoblastic leukemia (ALL) and 25 samples of acute myeloid leukemia (AML). ALL samples consist of two subtypes: 38 B-cell ALL and 9 T-cell ALL. The following two preprocessing steps are applied to dataset as in [40] 1) thresholding, the gene expression 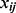 is set to 100 if 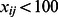 and set to 16000 if 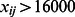; 2) filtering, the gene with 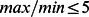 or 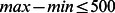 is excluded, where 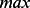 and 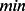 are the maximum and minimum expression levels for a particular gene across all the samples transformation.

For the leukemia dataset, a preliminary gene screening is used that the top 300 genes with the largest sample variances across all the samples are selected [40]. The model is fitted with 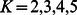, 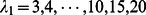, and 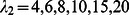.

#### Cancer subtype discovery

The clustering results of PMT-UC are compared with PMG-UC and PMT-DC. By a grid search, the optimal clustering results of three methods are shown in Table 5. PMT-DC can identify the 25 AML samples from all the 72 samples correctly. However, it cannot recognize the differences between two subtypes of ALL with the possible reason that PMT-DC assumes a diagonal covariance. Both PMT-UC and PMG-UC have better clustering performance than PMT-DC. The results in Table 5 clearly indicate that the robustness of PMT-UC make it perform better in identifying true clustering structures and gives a fewer errors in cancer subtype discovery.

**Table 5:** Optimal clustering results for the leukemia dataset.

	PMT-UC			PMG-UC			PMT-DC		
Clusters (#Samples)	1	2	3	1	2	3	1	2	3
ALL-B(38)	37	1	0	37	2	0	24	14	0
ALL-T(9)	0	8	1	0	8	1	8	0	1
AML(25)	1	0	24	2	0	24	0	0	25

#### Network structure analysis

**Figure 4:**
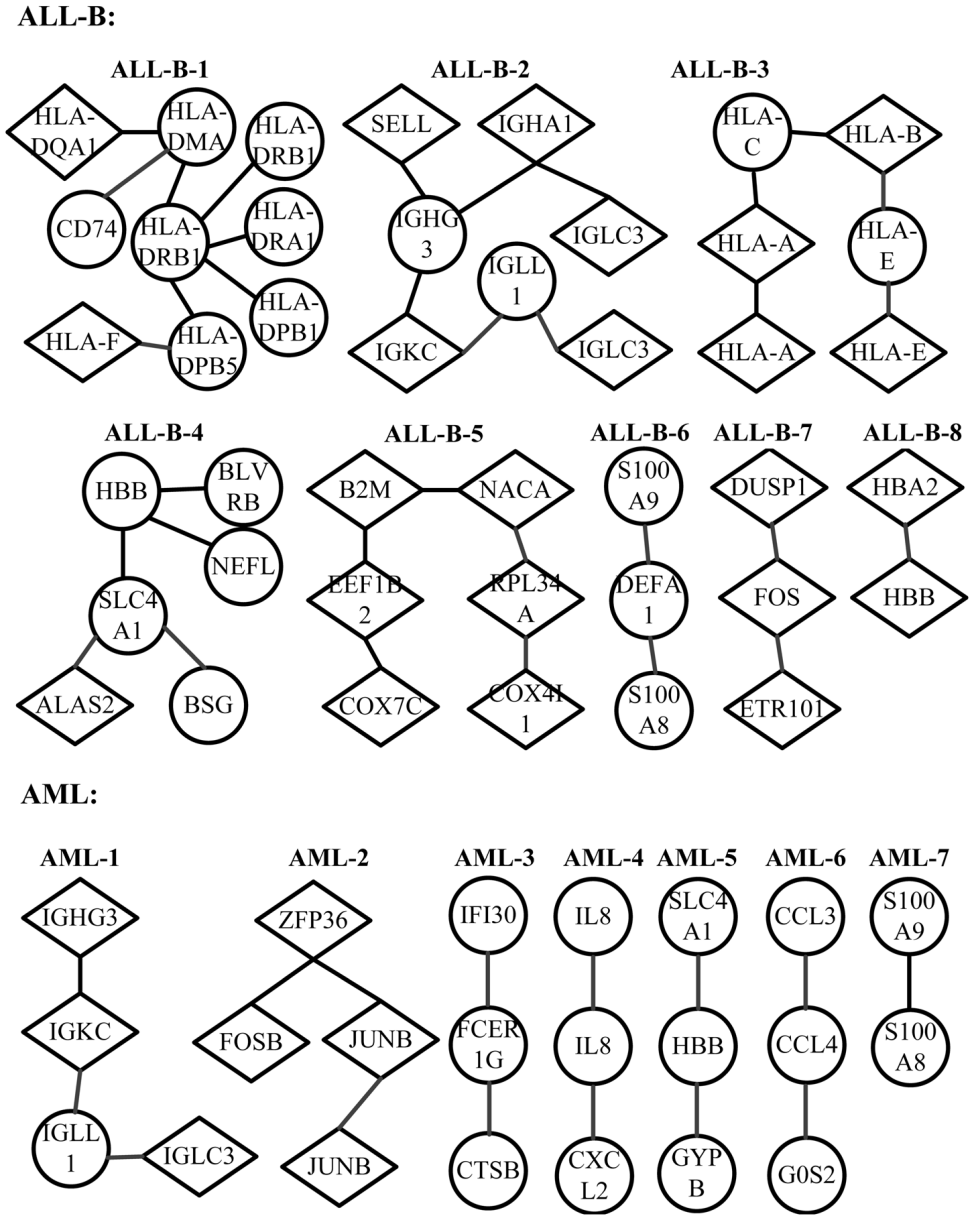
The subnetworks for ALL-B and AML of leukemia dataset estimated by PMT-UC. Nodes represent human genes, and they are connected by a link if their partial correlation derived from 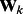 is larger than 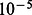. Each gene is labeled by its Gene Symbol (see Text S5 for the detailed information of the genes in each subnetwork). The shape of each node indicates whether the gene has cluster-specific means (circle) or not (diamond).

Figure 4 shows some example subnetworks based on the inverse scale matrices estimated by PMT-UC for ALL-B and AML. Each gene is labeled by its Gene Symbol. If the shape of the gene is circle, then it will have cluster-specific means, otherwise it will not have. For the ALL-T subtype, the related biomarkers identified by PMT-UC are all independent. It is shown that there are overlaps between some subnetworks corresponding to ALL-B and AML. However, these same genes interact with other different biomarkers under various cancer subtypes. Further more, the functional and biological relationships of the selected genes of each subnetwork are analyzed based on the GO annotation [41]. The P-value of a specific GO annotation is calculated using the hypergeometric distribution by the software GO::TermFinder [42]–[44]. Tables 6 and 7 list the GO analysis results for the subnetworks shown in Figure 4 of ALL-B and AML, respectively. The small P-value shows that the genes in each subnetwork have significant biological and functional correlation, and the common GO functions they share are often related to the subtypes of leukemia.

**Table 6:** The Gene Ontology results of the subnetwork for ALL-B of leukemia dataset.

Subnetwork	Elements	GO Number	Ontology Description	P-value
ALL-B-1	HLA-F HLA-DRB1 HLA-DRB5	GO:0071556	integral to lumenal side of endoplasmic	1.1×10^–20^
	CD74 HLA-DPB1 HLA-DPA1		reticulum membrane	
	HLA-DQA1 HLA-DRB1	GO:0012507	ER to Golgi transport vesicle membrane	1.1×10^–19^
ALL-B-1	CD74 HLA-DMA HLA-DRB1	GO:0019886	antigen processing and presentation of	2.8×10^–19^
	HLA-DRB5 HLA-DPB1 HLA-DPA1		exogenous peptide antigen via MHC class II	
	HLA-DQA1 HLA-DRB1	GO:0005765	lysosomal membrane	6.4×10^–18^
ALL-B-2	IGKC IGLC3 IGHG3 IGHA1	GO:0003823	antigen binding	6.2×10^–10^
ALL-B-2	IGKC IGLC3 IGHG3	GO:0006958	complement activation, classical pathway	1.6×10^–6^
ALL-B-3	The entire subnetwork	GO:0002474	antigen processing and presentation	1.3×10^–10^
			of peptide antigen via MHC class I	
ALL-B-4	HBB SLC4A1	GO:0015701	bicarbonate transport	6.8×10^–6^
ALL-B-4	ALAS2 BLVRB	GO:0042168	heme metabolic process	1.5×10^–5^
ALL-B-4	SLC4A1 NEFL	GO:0008022	protein C-terminus binding	8.0×10^–5^
ALL-B-5	EEF1B2 RPL35A	GO:0044444	cytoplasmic part	1.3×10^–4^
ALL-B-5	COX7C COX4I1	GO:0004129	cytochrome-c oxidase activity	1.9×10^–5^
ALL-B-6	The entire subnetwork	GO:0050832	defense response to fungus	1.5×10^–8^
		GO:0042742	defense response to bacterium	1.1×10^–5^
ALL-B-6	S100A9 S100A8	GO:0070488	neutrophil leukocyte aggregation	1.7×10^–7^
		GO:0002523	leukocyte migration involved in inflammatory response	3.7×10^–6^
ALL-B-7	DUSP1 FOS	GO:0051592	response to calcium ion	1.6×10^–3^
		GO:0051591	response to cAMP	1.6×10^–3^
ALL-B-8	The entire subnetwork	GO:0015671	oxygen transport	2.3×10^–6^
		GO:0031720	haptoglobin binding	2.1×10^–8^
		GO:0004601	peroxidase activity	4.3×10^–5^

The first column (Subnetwork) reports the name of the subnetwork introduced in Figure 4. The second column (Elements) presents the elements of subnetwork of which the functional and biological relationship are analyzed based on the GO annotation.

**Table 7:** The Gene Ontology results of the subnetwork for AML of leukemia dataset.

Subnetwork	Elements	GO Number	Ontology Description	P-value
AML-1	IGKC IGLC3 IGHG3	GO:0006958	complement activation, classical pathway	1.6×10^–6^
AML-2	FOSB JUNB	GO:0071277	cellular response to calcium ion	4.3×10^–5^
AML-3	IFI30 FCER1G	GO:0019886	antigen processing and presentation of	2.0×10^–4^
			exogenous peptide antigen via MHC class II	
		GO:0042590	antigen processing and presentation of	3.8×10^–4^
			exogenous peptide antigen via MHC class I	
AML-3	FCER1G CTSB	GO:0009897	external side of plasma membrane	4.0×10^–4^
AML-3	IFI30 CTSB	GO:0043202	lysosomal lumen	6.2×10^–5^
AML-4	The entire subnetwork	GO:0008009	chemokine activity	1.4×10^–5^
AML-5	SLC4A1 HBB	GO:0015701	bicarbonate transport	6.1×10^–6^
AML-6	CCL3 CCL4	GO:0031730	CCR5 chemokine receptor binding	1.6×10^–7^
		GO:0031726	CCR1 chemokine receptor binding	1.6×10^–7^
AML-7	The entire network	GO:0070488	neutrophil leukocyte aggregation	1.7×10^–7^
		GO:0002523	leukocyte migration involved in inflammatory response	3.7×10^–6^

The first column (Subnetwork) reports the name of the subnetwork introduced in Figure 4. The second column (Elements) presents the elements of subnetwork of which the functional and biological relationship are analyzed based on the GO annotation.

Specifically, for ALL-B, the smallest P-value is 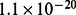 corresponding to GO:0071556 which is related to integral to lumenal side of endoplasmic reticulum membrane. All the genes in the subnetwork ALL-B-1 except HLA-DMA share this common GO function, including HLA-DQA1 and HLA-F which do not have cluster-specific means. HLA-DMA has high correlation with the genes in the same subnetwork in term of GO:0019886 and GO:0042613. The first term is also reported to be a significant GO function for leukemia in [45], which shows the general importance of antigen presentation and antigen processing for ALL. The second term is shown to be related with B-cells [46]. The subnetwork ALL-B-2 contains all the elements of the subnetwork AML-1. The genes IGKC, IGLC3, IGHG3, IGHA1 share the common GO function GO:0003823 which is related with B cell receptor activity. In addition, the IGLL1 gene encodes Lambda5, a component of the pre-B cell receptor (pre-BCR) which plays an important role in acute lymphoblastic leukemia [47], [48]. The genes IGKC, IGLC3, IGHG3 shared by two subnetworks have the common GO function GO:0006958 which is inferred to be part of GO:0002443 (leukocyte mediated immunity). In ALL-B-2, SELL is also associated with leukocyte that mediates leukocyte rolling and leukocyte adhesion to endothelium at sites of inflammation [49]. The term GO:0002474 shared by the subnetwork ALL-B-3 is related to antigen presentation and has been reported to be highly statistically significant in subtypes of ALL [50].

Next, the subnetworks of which all the elements are not mean-based discriminative genes are taken account into. In subnetwork ALL-T-5, COX7C and COX4I1 which have common GO function cytochrome-c oxidase activity are indirectly connected by other elements. The genes are essential for maintaining the integrity of the subnetwork. HBB and HBA2 in ALL-T-8 have the function of binding with oxygen molecules and transporting them to the blood stream. They are shown to be correlated with various kinds of cancers [51]. The genes ZFP36, FOSB, JUNB belonging to AML-2 are transcription factors whose dysregulation is essential for leukemic stem cell function and that are targets for therapeutic interventions [52].

#### Biomarker identification

The gene selection results and biological meanings of the biomarkers selected by PMT-UC are presented in this section (see Text S6 for the detailed information of all the selected biomarkers). There are 210 genes selected as informative, including 161 mean-based discriminative genes. Most of identified biomarkers are considered to have diagnostic values for leukemia. For example, CST3 is identified as a validated target for investigating the basic biology of ALL and AML [53]. CD74 has been shown to be associated with B cell lymphocytic leukemia cell survival [54]. MPO is a lysosomal enzyme highly expressed in bone marrow cells and has been reported to be associated with risk of acute lymphoblastic leukemia [55]. TCL1A expression has been shown to delineate biological and clinical variability in B-cell lymphoma and can be regarded as a potential therapeutic target [56]. It has been shown that increased levels of LYZ in urine and serum are diagnostic indicators for some kinds of leukemia [57].

Unlike conventional penalized model-based clustering, our network-based gene selection criterion can implicate disease-related genes with low discriminative potential, such as MIF, ANP32B, METAP2, SOX4. MIF is shown to recognize the CD74 extracellular domain as a cell surface receptor, and also be associated with B cell lymphocytic leukemia cell survival as CD74 [54]. ANP32B is acted as a negative regulator for leukemic cell apoptosis and may serve as a potential therapeutic target for leukemia treatment [58], [59]. METAP2 has been detected to have high levels in B-cell acute lymphoblastic leukemia derived from germinal center B cells [60]. SOX4 has been proven to enable oncogenic survival signals in acute lymphoblastic leukemia recently [61].

### Conclusions

A new robust penalized model-based network clustering for cancer subtype discovery, underlying network reconstruction and network-based biomarker identification is proposed. The multivariate Student’s t distribution used for the components of the mixture model results in robust clustering assignment. It permits a treatment of unconstrained covariance matrices to take gene dependencies into account. The network-based gene selection criterion we proposed can find the genes which have low discriminative potential, but interact with discriminative genes or have cluster-specific underlying network structures. This property is important for the discovery of disease-causing genes, because the phenotypic changes for some cancers do not regulate the level of expression.

The results for binary-clusters simulation studies have demonstrated the utility of the proposed method and its superior clustering and gene selection performance over penalized model-based Gaussian clustering with unconstrained covariance (PMG-UC) and penalized model-based Student’s t clustering with diagonal covariance (PMT-DC). Compared with PMG-UC, the network reconstruction results show that our algorithm can still discover the relationship between genes under the network even if the datasets have high noise.

The algorithm has been also applied for the analysis of a large data set consisting of leukemia cancer subtypes. The comparison of the clustering results for the three methods demonstrates that our method can handle the outliers and identify the cancer subtypes with different underlying networks or pathways. The most selected biomarkers have biological meanings and are proven to be related with leukemia. The functional and biological correlation of the genes in the same subnetwork is analyzed based on the GO annotation. The significant interaction between the genes can provide basis for the establishment of large relational network database.

Since the EM algorithm for PMT-UC is based on graphical lasso which is not feasible with high dimension, we need to apply preprocessing steps to filter some genes, which may result in the missing of the informative biomarkers. Therefore, in the future work, more efficient algorithms that can handle high-dimensional dataset are needed for the accuracy of gene selection. Moreover, the multiple-clusters simulation experiment indicates that PMT-UC should be used with caution when the dataset has more thin-tailed clusters of which some ones may do not have enough mean-based discriminative genes. The flexibility of PMT-UC may make it explain the extra clusters by fat tails. With the availability of genetic pathways or networks for genes under various conditions, we can incorporate these sources as prior information into building gene expression-based clustering and variable selection methods. They will facilitate the discovery of the true underlying clusters and biomarkers.

## Supporting Information

Text S1 **The penalized log-likelihood of the complete data in the EM algorithm for PMT-UC.**
(PDF)

Text S2 **Computation of the expectation of the penalized log-likelihood of the complete data for PMT-UC.**
(PDF)

Text S3
**The updating estimate of the local parameter 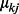.**
(PDF)

Text S4
**The results for network reconstruction of the total 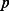 genes of the simulated datasets.**
(PDF)

Text S5 **The detailed information of genes in the subnetworks corresponding to B-cell ALL and AML for leukemia dataset.**
(PDF)

Text S6 **The detailed information of all the leukemia-related biomarkers selected by PMT-UC.**
(PDF)
