# Supplementary material for: Cancer Subtype Discovery and Biomarker Identification via a New Robust Network Clustering Algorithm
Source: PLoS One. 2013 Jun 17;8(6):e66256. doi: 10.1371/journal.pone.0066256 (PMC3684607; doi:10.1371/journal.pone.0066256)
Supplement: Text S1 — The penalized log-likelihood of the complete data in the EM algorithm for PMT-UC. (PDF) [file pone.0066256.s001.pdf]

The penalized log-likelihood of the complete data in the EM algorithm for PMT-UC

In EM algorithm, we assume that each sample $\mathbf{x}_i$ has a corresponding unobserved indicator vector $\mathbf{z}_i = [z_{i1}\ z_{i2}\ \cdots\ z_{iK}]$, specifying the mixture component that $\mathbf{x}_i$ belongs to. If $\mathbf{x}_i$ comes from component k then $z_{ik} = 1$, otherwise $z_{ik} = 0$. Given $z_{ik} = 1$, $\mathbf{x}_i$ follows a Student's t distribution with the probability density function $f_k(\mathbf{x}_i; \theta_k)$. According to the fact that the Student's t distribution can be written as a multivariate Gaussian distribution with the covariance matrix scaled by the reciprocal of a Gamma random variable, the additional missing data $\mathbf{u}_i = [u_{i1}\ u_{i2}\ \cdots\ u_{iK}]$ is introduced, where each element u_{ik} of $\mathbf{u}_i$ follows the Gamma distribution. Then we have the relationship between $\mathbf{z}_i$, $\mathbf{u}_i$, and $\mathbf{x}_i$ as follows:

$$\begin{aligned}
&\mathbf{z}_i \sim \text{Multinomial}(\pi_1, \cdots, \pi_K),\\
&u_{ik} | z_{ik} = 1 \sim \text{Gamma}\left(\frac{1}{2}\nu_k, \frac{1}{2}\nu_k\right),\\
&\mathbf{x}_i | u_{ik}, z_{ik} = 1 \sim N\left(\mu_k, \frac{\boldsymbol{\Sigma}_k}{u_{ik}}\right),
\end{aligned}$$

where the probability density function $f^M(\mathbf{z}; \pi_1, \cdots, \pi_K)$ of Multinomial$(\pi_1, \cdots, \pi_K)$ is given by

$$f^M(\mathbf{z}; \pi_1, \cdots, \pi_K) = \pi_1^{z_1} \pi_2^{z_2} \cdots \pi_K^{z_K},$$

the probability density function $f^G(u; \frac{1}{2}\nu_k, \frac{1}{2}\nu_k)$ of Gamma$(\frac{1}{2}\nu_k, \frac{1}{2}\nu_k)$ is given by

$$f^G\left(u; \frac{1}{2}\nu_k, \frac{1}{2}\nu_k\right) = \frac{\nu_k^{\nu_k/2}}{2^{\nu_k/2}\Gamma(\nu_k/2)} u^{\nu_k/2-1} \exp\left(-\frac{\nu_k}{2}u\right),$$

and the probability density function $f^N\left(\mathbf{x}; \mu_k, \frac{\boldsymbol{\Sigma}_k}{u_{ik}}\right)$ of $N\left(\mu_k, \frac{\boldsymbol{\Sigma}_k}{u_{ik}}\right)$ is given by

$$f^N\left(\mathbf{x}; \mu_k, \frac{\boldsymbol{\Sigma}_k}{u_{ik}}\right) = (\boldsymbol{\Sigma}_k / u_{ik})^{-\frac{1}{2}} (2\pi)^{-\frac{p}{2}} \times \exp\left[-\frac{u_{ik}}{2}(\mathbf{x} - \mu_k)' \boldsymbol{\Sigma}_k^{-1} (\mathbf{x} - \mu_k)\right].$$

The complete data becomes $x_i^c = (\mathbf{x}_i, \mathbf{z}_i, \mathbf{u}_i)$ of which the probability density function is [1]:

$$f^c(x_i^c; \Psi) = \prod_{k=1}^{K} f_k(\mathbf{x}_i; \theta_k)^{z_{ik}} = \prod_{k=1}^{K} \left[\pi_k f^G\left(u_{ik}; \frac{1}{2}\nu_k, \frac{1}{2}\nu_k\right) f^N\left(\mathbf{x}_i; \mu_k, \frac{\boldsymbol{\Sigma}_k}{u_{ik}}\right)\right]^{z_{ik}}. \tag{S1}$$

Then, the complete data penalized log-likelihood can be divided into four parts:

$$\begin{aligned}
l_{\text{c,pen}}(\Psi) &= \sum_{i=1}^{n} \log f^c(x_i^c; \Psi) - \text{pen}_\lambda(\Phi)\\
&= \sum_{i=1}^{n}\sum_{k=1}^{K} z_{ik} \log\left[\pi_k f_k(\mathbf{x}_i; \theta_k)\right] - \text{pen}_\lambda(\Phi)\\
&= \sum_{i=1}^{n}\sum_{k=1}^{K} z_{ik} \left[\log \pi_k + \log f^G\left(u_{ik}; \frac{1}{2}\nu_k, \frac{1}{2}\nu_k\right) + \log f^N\left(\mathbf{x}_i; \mu_k, \frac{\boldsymbol{\Sigma}_k}{u_{ik}}\right)\right] - \text{pen}_\lambda(\Phi)\\
&\triangleq l_1(\pi) + l_2(\nu) + l_3(\Phi) - \text{pen}_\lambda(\Phi),
\end{aligned} \tag{S2}$$

where

$$l_1(\pi) = \sum_{i=1}^{n} \sum_{k=1}^{K} z_{ik} \log \pi_k, \tag{S3}$$

$$l_2(\nu) = \sum_{i=1}^{n} \sum_{k=1}^{K} z_{ik} \left[-\log \Gamma\left(\frac{\nu_k}{2}\right) + \frac{\nu_k}{2} \log\left(\frac{\nu_k}{2}\right) + \frac{\nu_k}{2}(\log u_{ik} - u_{ik}) - \log u_{ik} \right], \tag{S4}$$

and

$$l_3(\Phi) = \sum_{i=1}^{n} \sum_{k=1}^{K} \left[-\frac{p}{2} \log(2\pi) + \frac{p}{2} \log u_{ik} + \frac{\log |\mathbf{W}_k|}{2} - \frac{u_{ik}(\mathbf{x}_i - \mu_k)' \mathbf{W}_k (\mathbf{x}_i - \mu_k)}{2} \right], \tag{S5}$$

with $\mathbf{W}_k = \mathbf{\Sigma}_k^{-1}$.

REFERENCES

[1] C. Fraley and A. E. Raftery. Model-based clustering, discriminant analysis, and density estimation. *J Am Stat Assoc*, 97(458):611–631, 2002.
