# Supplementary material for: Cancer Subtype Discovery and Biomarker Identification via a New Robust Network Clustering Algorithm
Source: PLoS One. 2013 Jun 17;8(6):e66256. doi: 10.1371/journal.pone.0066256 (PMC3684607; doi:10.1371/journal.pone.0066256)
Supplement: Text S2 — Computation of the expectation of the penalized log-likelihood of the complete data for PMT-UC. (PDF) [file pone.0066256.s002.pdf]

Computation of the expectation of the penalized log-likelihood of the complete data for PMT-UC

The expected value $Q(\Psi;\Psi^{(t)})$ of $l_{\text{c,pen}}(\Psi)$ with respect to the current estimation $\Psi^{(t)}$ of the parameters at the tth iteration is as follows:

$$\begin{aligned} Q(\Psi;\Psi^{(t)}) &= E_{\Psi^{(t)}}\{l_{\text{c,pen}}(\Psi)|\mathcal{X}\} \\ &= E_{\Psi^{(t)}}\{l_1(\pi)|\mathcal{X}\} + E_{\Psi^{(t)}}\{l_2(\nu)|\mathcal{X}\} + E_{\Psi^{(t)}}\{l_3(\Phi)|\mathcal{X}\} - \text{pen}_\lambda(\Phi) \\ &\triangleq Q_1(\pi;\Psi^{(t)}) + Q_2(\nu;\Psi^{(t)}) + Q_3(\Phi;\Psi^{(t)}) - \text{pen}_\lambda(\Phi) \qquad \text{(S6)} \\ &\triangleq Q_1(\pi;\Psi^{(t)}) + Q_2(\nu;\Psi^{(t)}) + Q_{3,\text{pen}}(\Phi;\Psi^{(t)}), \qquad \text{(S7)} \end{aligned}$$

According to Text S1 and (S6), in order to calculate $Q(\Psi;\Psi^{(t)})$, we need to get the value of $E_{\Psi^{(t)}}(z_{ik}|\mathbf{x}_i)$, $E_{\Psi^{(t)}}(u_{ik}|\mathbf{x}_i, z_{ik}=1)$ and $E_{\Psi^{(t)}}(\log u_{ik}|\mathbf{x}_i, z_{ik}=1)$ for $i=1,\cdots,n$, $k=1,\cdots,K$.

Since $\mathbf{x}_i$ comes from the mixture distribution with probability density function $g(\mathbf{x}_i;\Psi)$ and $\mathbf{z}_i$ follows the Multinomial distribution with probability density function $f^M(\mathbf{z};\pi_1,\cdots,\pi_K)$, the value of $E_{\Psi^{(t)}}(z_{ik}|\mathbf{x}_i)$ is given by

$$E_{\Psi^{(t)}}(z_{ik}|\mathbf{x}_i) = \frac{\pi_k^{(t)} f_k(\mathbf{x}_i;\theta_k^{(t)})}{g(\mathbf{x}_i;\Psi^{(t)})} \triangleq \tau_{ik}^{(t)}. \qquad \text{(S8)}$$

$\tau_{ik}^{(t)}$ can be regarded as the posterior probability of $\mathbf{x}_i$ belonging to the kth cluster. Seeing that the Gamma distribution is conjugate to itself (self-conjugate) with respect to a Gaussian likelihood function, we have

$$E_{\Psi^{(t)}}(u_{ik}|\mathbf{x}_i, z_{ik}=1) = \frac{\nu_k^{(t)}+p}{\nu_k^{(t)}+\delta(\mathbf{x}_i;\mu_k^{(t)},\mathbf{\Sigma}_k^{(t)})} \triangleq u_{ik}^{(t)}, \qquad \text{(S9)}$$

and

$$E_{\Psi^{(t)}}(\log u_{ik}|\mathbf{x}_i, z_{ik}=1) = \log u_{ik}^{(t)} + \psi\left(\frac{\nu_k^{(t)}+p}{2}\right) - \log\left(\frac{\nu_k^{(t)}+p}{2}\right), \qquad \text{(S10)}$$

where $\psi(s) = \{\partial\Gamma(s)/\partial s\}/\Gamma(s)$ is the Digamma function [1].

Based on (S8), (S9) and (S10), after deleting the terms not related with the corresponding parameters, the first three parts of (S6) can be expressed as

$$Q_1(\pi;\Psi^{(t)}) = E_{\Psi^{(t)}}\{l_1(\pi)|\mathcal{X}\} = \sum_{i=1}^n\sum_{k=1}^K \log\pi_k E_{\Psi^{(t)}}\{z_{ik}|\mathbf{x}_i\} = \sum_{i=1}^n\sum_{k=1}^K \tau_{ik}^{(t)}\log\pi_k, \qquad \text{(S11)}$$

$$\begin{aligned} Q_2(\nu;\Psi^{(t)}) &= E_{\Psi^{(t)}}\{l_2(\nu)|\mathcal{X}\} \\ &= \sum_{i=1}^n\sum_{k=1}^K \Big[-\log\Gamma\left(\frac{\nu_k}{2}\right) + \frac{\nu_k}{2}\log\left(\frac{\nu_k}{2}\right) - \frac{\nu_k}{2}E_{\Psi^{(t)}}\{u_{ik}|\mathbf{x}_i\} \\ &+ \left(\frac{\nu_k}{2}-1\right)E_{\Psi^{(t)}}\{\log u_{ik}|\mathbf{x}_i\}\Big] E_{\Psi^{(t)}}\{z_{ik}|\mathbf{x}_i\} \\ &= \sum_{i=1}^n\sum_{k=1}^K \tau_{ik}^{(t)}\Big\{-\log\Gamma\left(\frac{\nu_k}{2}\right) + \frac{\nu_k}{2}\log\left(\frac{\nu_k}{2}\right) + \frac{\nu_k}{2}\Big[\log u_{ik}^{(t)} \\ &- u_{ik}^{(t)} + \psi\left(\frac{\nu_k^{(t)}+p}{2}\right) - \log\left(\frac{\nu_k^{(t)}+p}{2}\right)\Big]\Big\}, \qquad \text{(S12)} \end{aligned}$$

and

$$
\begin{aligned}
Q_3(\Phi;\Psi^{(t)}) &= E_{\Psi^{(t)}}\{l_3(\Phi)|\mathcal{X}\} \\
&= \sum_{i=1}^{n}\sum_{k=1}^{K}\left[-\frac{p}{2}\log(2\pi)+\frac{1}{2}\log|\mathbf{W}_k|+\frac{p}{2}\log u_{ik}^{(t)}\right. \\
&- \left.\frac{u_{ik}^{(t)}}{2}(\mathbf{x}_i-\mu_k)'\mathbf{W}_k(\mathbf{x}_i-\mu_k)\right]E_{\Psi^{(t)}}\{z_{ik}|\mathbf{x}_i\} \\
&= \sum_{i=1}^{n}\sum_{k=1}^{K}\tau_{ik}^{(t)}\left[-\frac{p}{2}\log(2\pi)+\frac{1}{2}\log|\mathbf{W}_k|\right. \\
&+ \left.\frac{p}{2}\log u_{ik}^{(t)}-\frac{u_{ik}^{(t)}}{2}(\mathbf{x}_i-\mu_k)'\mathbf{W}_k(\mathbf{x}_i-\mu_k)\right]. \qquad \text{(S13)}
\end{aligned}
$$

References

[1] D. Peel and G. J. McLachlan. Robust mixture modelling using the t distribution. *Stat Comput*, 10(4):339–348, 2000.
