# Supplementary material for: Cancer Subtype Discovery and Biomarker Identification via a New Robust Network Clustering Algorithm
Source: PLoS One. 2013 Jun 17;8(6):e66256. doi: 10.1371/journal.pone.0066256 (PMC3684607; doi:10.1371/journal.pone.0066256)
Supplement: Text S3 — The updating estimate of the local parameter . (PDF) [file pone.0066256.s003.pdf]

The updating estimate of the local parameter μ_{kj}

The objective function for the updating of μ_{kj} is:

$$Q_4(\mu) = \sum_{i=1}^{n}\sum_{k=1}^{K}\tau_{ik}\left[-\frac{u_{ik}}{2}(\mathbf{x}_i-\mu_k)'\mathbf{W}_k(\mathbf{x}_i-\mu_k)\right] - \lambda_1\sum_{k=1}^{K}\sum_{j=1}^{p}\omega_{kj}|\mu_{kj}|. \tag{S14}$$

We consider the following two cases [1]:

- If $\mu_{kj} \neq 0$ is a maximum, since $Q_4(\mu)$ is concave and differentiable, the global maximum of $Q_4(\mu)$ can be get by setting $\partial Q_4(\mu)/\partial\mu_{kj} = 0$. Therefore, the updating estimate μ_{kj} is the solution of the following equation

$$\sum_{i=1}^{n}\tau_{ik}u_{ik}\left[\sum_{q=1}^{p}(x_{il}-\mu_{kq})W_{k,jq}\right] - \lambda_1\omega_{kj}\text{sign}(\mu_{kj}) = 0. \tag{S15}$$

- If $\mu_{kj} = 0$ is a maximum, then the value of $Q_4(\mu)$ at $\mu_{kj} = 0$ will bigger than that at any $\Delta\mu_{kj}$ near 0 while other components of μ_k are fixed at its maximum. Therefore, we have

$$\begin{aligned}
&\sum_{i=1}^{n}\tau_{ik}u_{ik}\Big[(\mathbf{x}_i-\mu_k)'\mathbf{W}_k(\mathbf{x}_i-\mu_k)\ |_{\mu_{kj}=\Delta\mu_{kj}} \\
&- (\mathbf{x}_i-\mu_k)'\mathbf{W}_k(\mathbf{x}_i-\mu_k)\ |_{\mu_{kj}=0}\Big] \geq -2\lambda_1\omega_{kj}|\Delta\mu_{kj}|. \\
\Leftrightarrow\quad &\sum_{i=1}^{n}\tau_{ik}u_{ik}\left[2\Delta\mu_{kj}\sum_{q=1,q\neq j}^{p}(x_{iq}-\mu_{kq})W_{k,jq} + W_{k,jj}(-\Delta\mu_{kj}^2 + 2x_{ij}\Delta\mu_{kj})\right] \leq 2\lambda_1\omega_{kj}|\Delta\mu_{kj}| \\
\Leftrightarrow\quad &\left|\frac{1}{\omega_{kj}}\sum_{i=1}^{n}\tau_{ik}u_{ik}\left[\sum_{q=1,q\neq j}^{p}(x_{iq}-\mu_{kq})W_{k,jq} + x_{ij}W_{k,jj}\right]\right| \leq \lambda_1, \text{as } \Delta\mu_{kj}\to 0
\end{aligned}$$

Hence, we have the below updating formula for the local parameter: if

$$\left|\frac{1}{\omega_{kj}^{(t)}}\sum_{i=1}^{n}\tau_{ik}^{(t)}u_{ik}^{(t)}\left(\sum_{q=1,q\neq j}^{p}(x_{iq}-\mu_{kq}^{(t)})W_{k,jq}^{(t)} + x_{ij}W_{k,jj}^{(t)}\right)\right| \leq \lambda_1, \tag{S16}$$

then $\mu_{kj}^{(t+1)} = 0$; otherwise

$$\left(\sum_{i=1}^{n}\tau_{ik}^{(t)}u_{ik}^{(t)}\right)\mu_{kj}^{(t+1)}W_{k,jj}^{(t)} + \lambda_1\omega_{kj}^{(t)}\text{sign}\left(\mu_{kj}^{(t+1)}\right) = \sum_{i=1}^{n}\tau_{ik}^{(t)}u_{ik}^{(t)}\left(\mathbf{x}_i\mathbf{W}_{k,.j}^{(t)} - \mu_k^{(t)}\mathbf{W}_{k,.j}^{(t)} - \mu_{kj}^{(t)}W_{k,jj}^{(t)}\right). \tag{S17}$$

References

[1] H. Zhou, W. Pan, and X. Shen. Penalized model-based clustering with unconstrained covariance matrices. *Electron J Stat*, 3:1473–1496, 2009.
