# Supplementary material for: Cancer Subtype Discovery and Biomarker Identification via a New Robust Network Clustering Algorithm
Source: PLoS One. 2013 Jun 17;8(6):e66256. doi: 10.1371/journal.pone.0066256 (PMC3684607; doi:10.1371/journal.pone.0066256)
Supplement: Text S4 — The results for network reconstruction of the total genes of the simulated datasets. (PDF) [file pone.0066256.s004.pdf]

The results for network reconstruction of the total p genes of the simulated datasets

In this section, we show the true cluster-specific sparsity patterns of the total p genes and those inferred by PMT-UC and PMG-UC for the four set-ups of the three cases when $\nu = 20, 10, 6$.

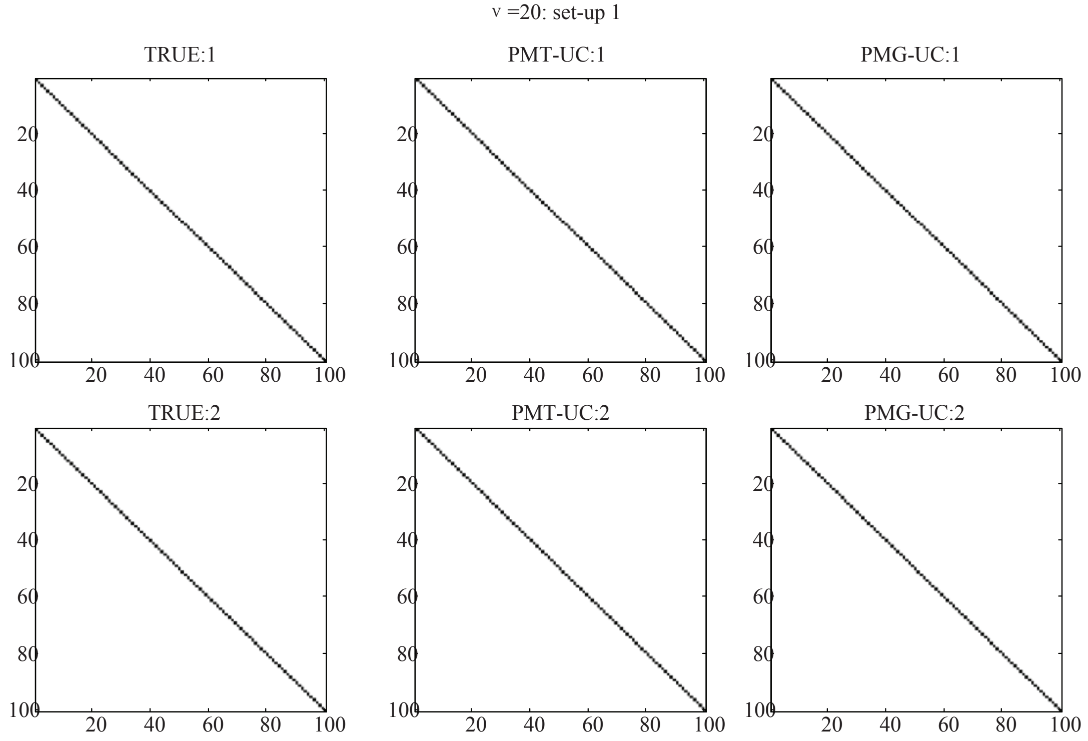

Fig. S1. Network reconstruction for simulated dataset in set-up 1 when $\nu = 20$. TRUE:1 and TRUE:2 are the original scale matrices $\boldsymbol{W}_1$ and $\boldsymbol{W}_2$ for the first and second clusters, respectively. PMT-UC:1 and PMT-UC:2 are the estimation of the scale matrices using PMT-UC. PMG-UC:1 and PMG-UC:2 are the estimation of the covariance matrices using PMG-UC.

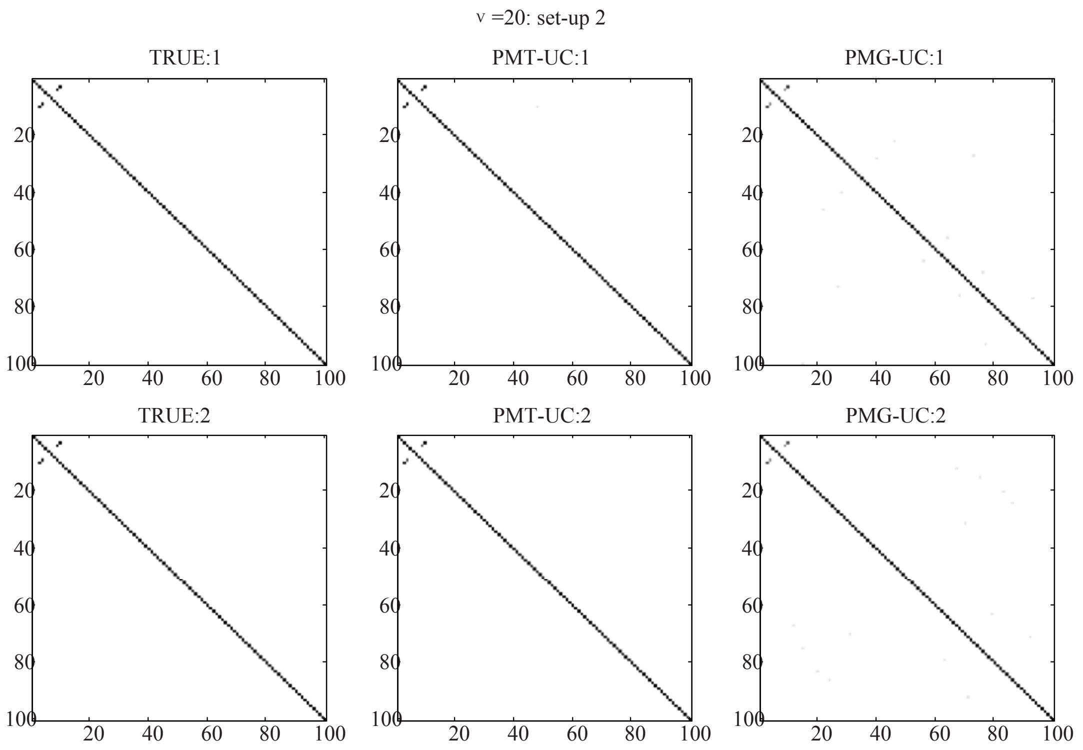

Fig. S2. Network reconstruction for simulated dataset in set-up 2 when $\nu = 20$. TRUE:1 and TRUE:2 are the original scale matrices $\boldsymbol{W}_1$ and $\boldsymbol{W}_2$ for the first and second clusters, respectively. PMT-UC:1 and PMT-UC:2 are the estimation of the scale matrices using PMT-UC. PMG-UC:1 and PMG-UC:2 are the estimation of the covariance matrices using PMG-UC.

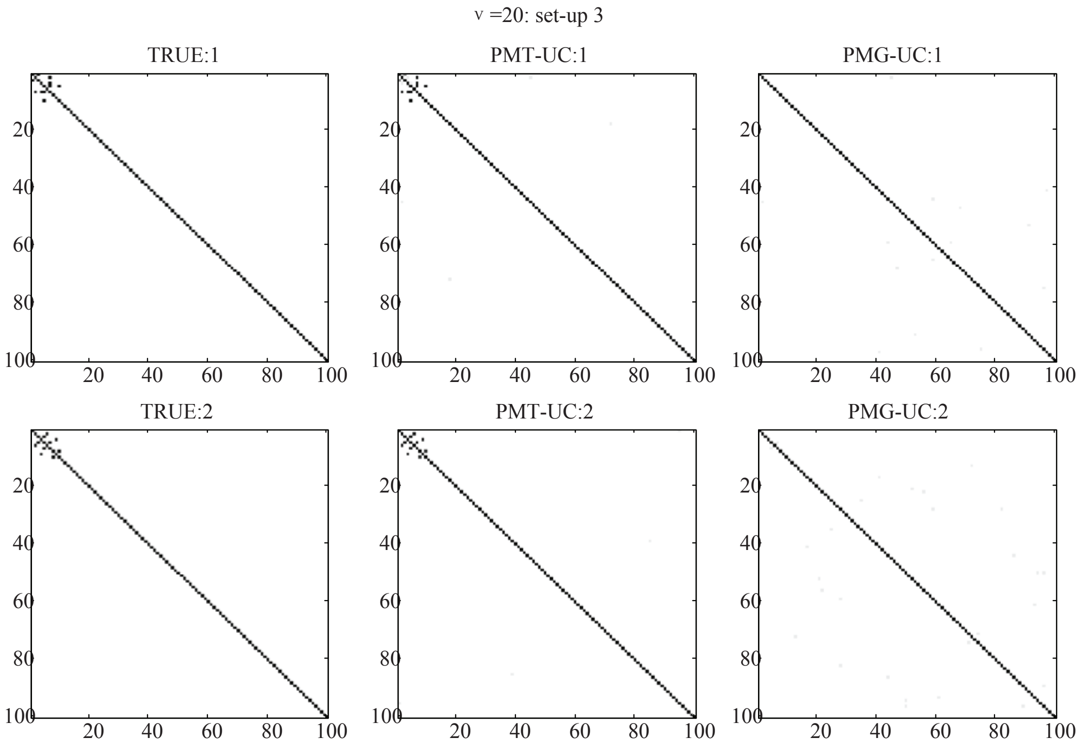

Fig. S3. Network reconstruction for simulated dataset in set-up 3 when $\nu = 20$. TRUE:1 and TRUE:2 are the original scale matrices $\boldsymbol{W}_1$ and $\boldsymbol{W}_2$ for the first and second clusters, respectively. PMT-UC:1 and PMT-UC:2 are the estimation of the scale matrices using PMT-UC. PMG-UC:1 and PMG-UC:2 are the estimation of the covariance matrices using PMG-UC.

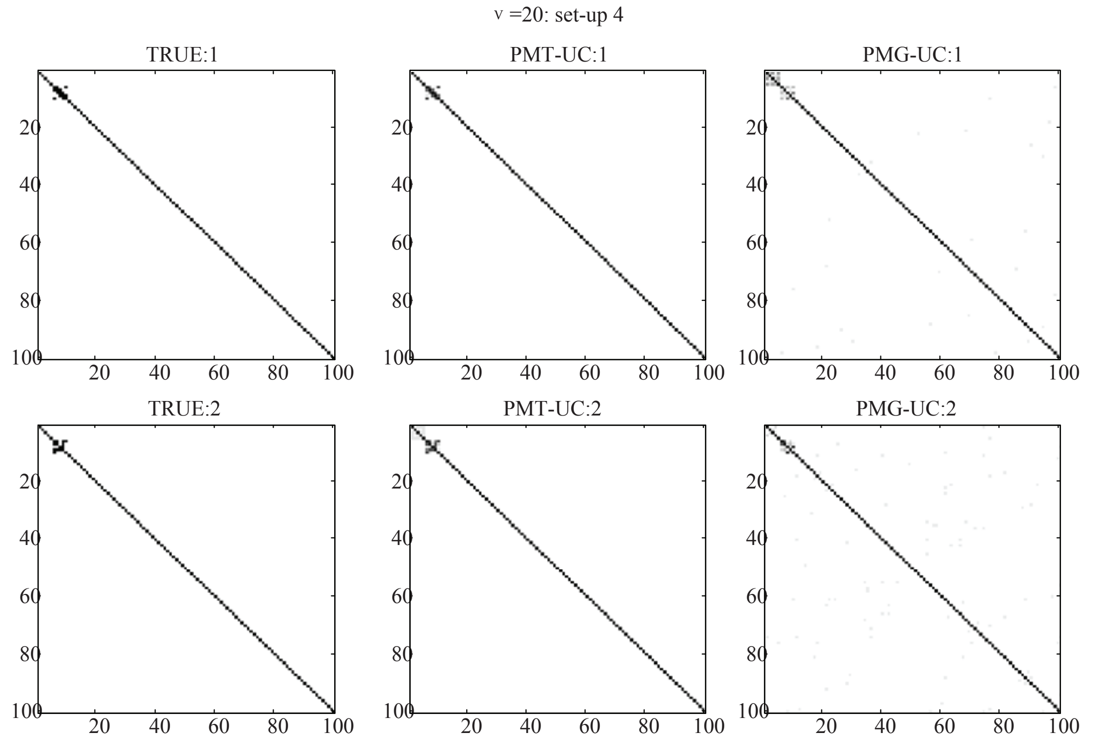

Fig. S4. Network reconstruction for simulated dataset in set-up 4 when $\nu = 20$. TRUE:1 and TRUE:2 are the original scale matrices $\boldsymbol{W}_1$ and $\boldsymbol{W}_2$ for the first and second clusters, respectively. PMT-UC:1 and PMT-UC:2 are the estimation of the scale matrices using PMT-UC. PMG-UC:1 and PMG-UC:2 are the estimation of the covariance matrices using PMG-UC.

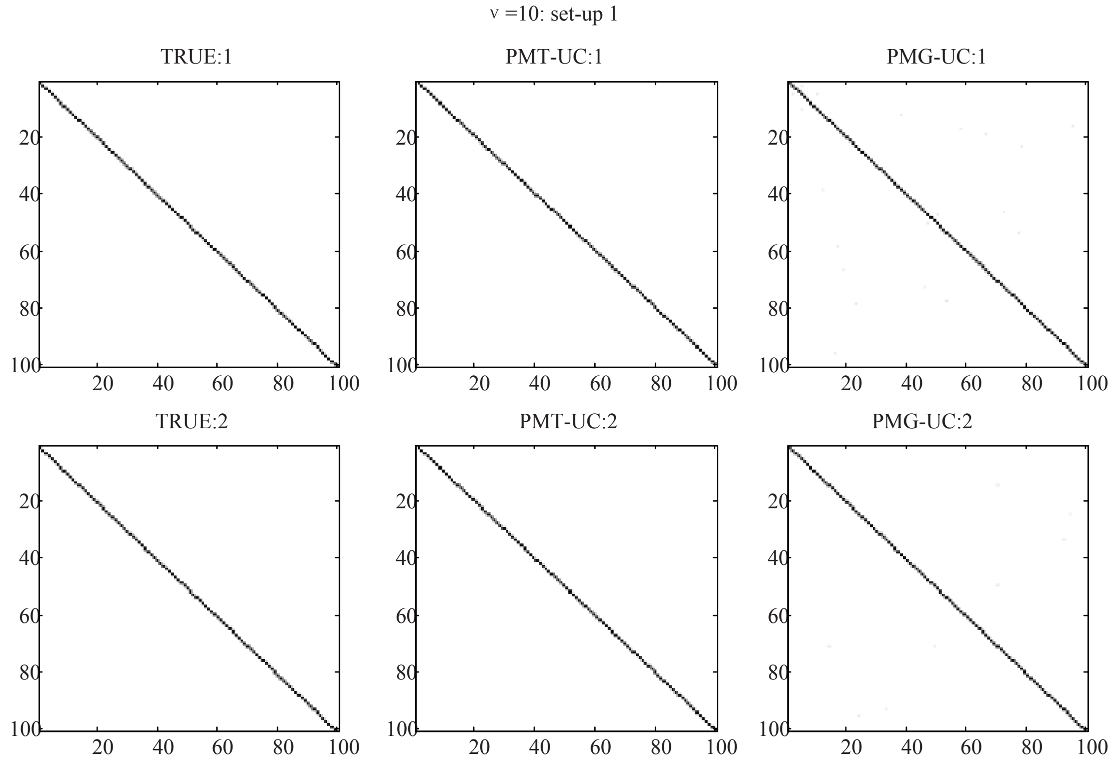

Fig. S5. Network reconstruction for simulated dataset in set-up 1 when $\nu = 10$. TRUE:1 and TRUE:2 are the original scale matrices $\boldsymbol{W}_1$ and $\boldsymbol{W}_2$ for the first and second clusters, respectively. PMT-UC:1 and PMT-UC:2 are the estimation of the scale matrices using PMT-UC. PMG-UC:1 and PMG-UC:2 are the estimation of the covariance matrices using PMG-UC.

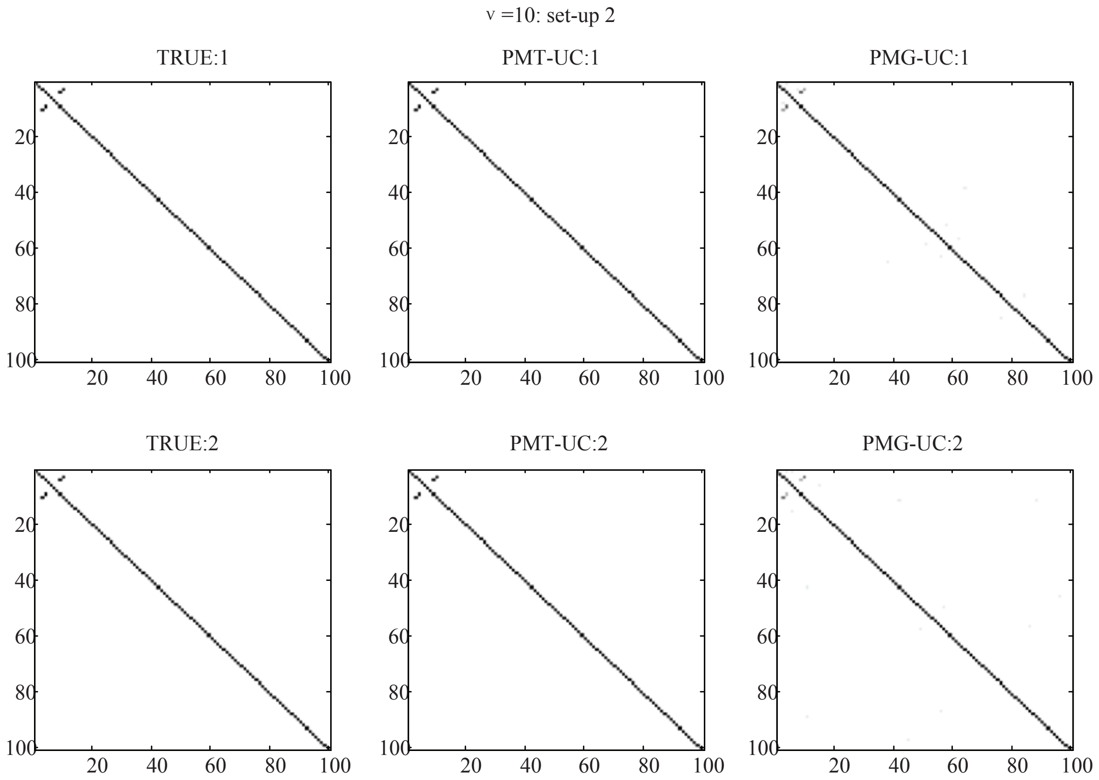

Fig. S6. Network reconstruction for simulated dataset in set-up 2 when $\nu = 10$. TRUE:1 and TRUE:2 are the original scale matrices $\boldsymbol{W}_1$ and $\boldsymbol{W}_2$ for the first and second clusters, respectively. PMT-UC:1 and PMT-UC:2 are the estimation of the scale matrices using PMT-UC. PMG-UC:1 and PMG-UC:2 are the estimation of the covariance matrices using PMG-UC.

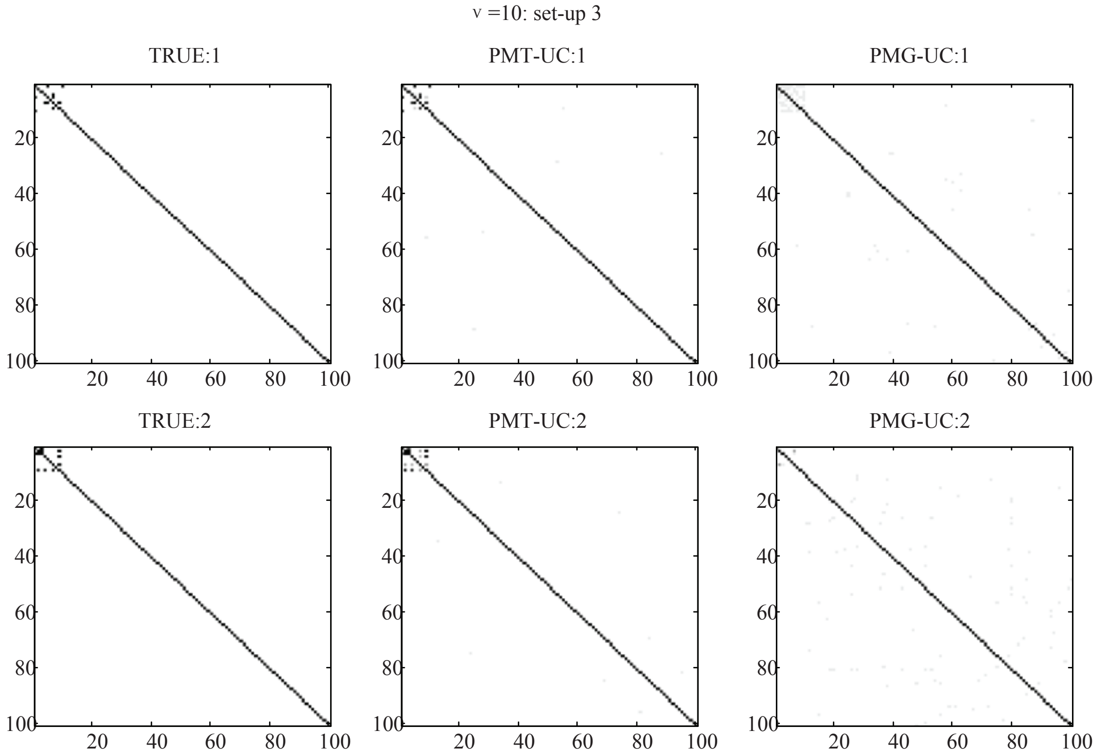

Fig. S7. Network reconstruction for simulated dataset in set-up 3 when $\nu = 10$. TRUE:1 and TRUE:2 are the original scale matrices $\boldsymbol{W}_1$ and $\boldsymbol{W}_2$ for the first and second clusters, respectively. PMT-UC:1 and PMT-UC:2 are the estimation of the scale matrices using PMT-UC. PMG-UC:1 and PMG-UC:2 are the estimation of the covariance matrices using PMG-UC.

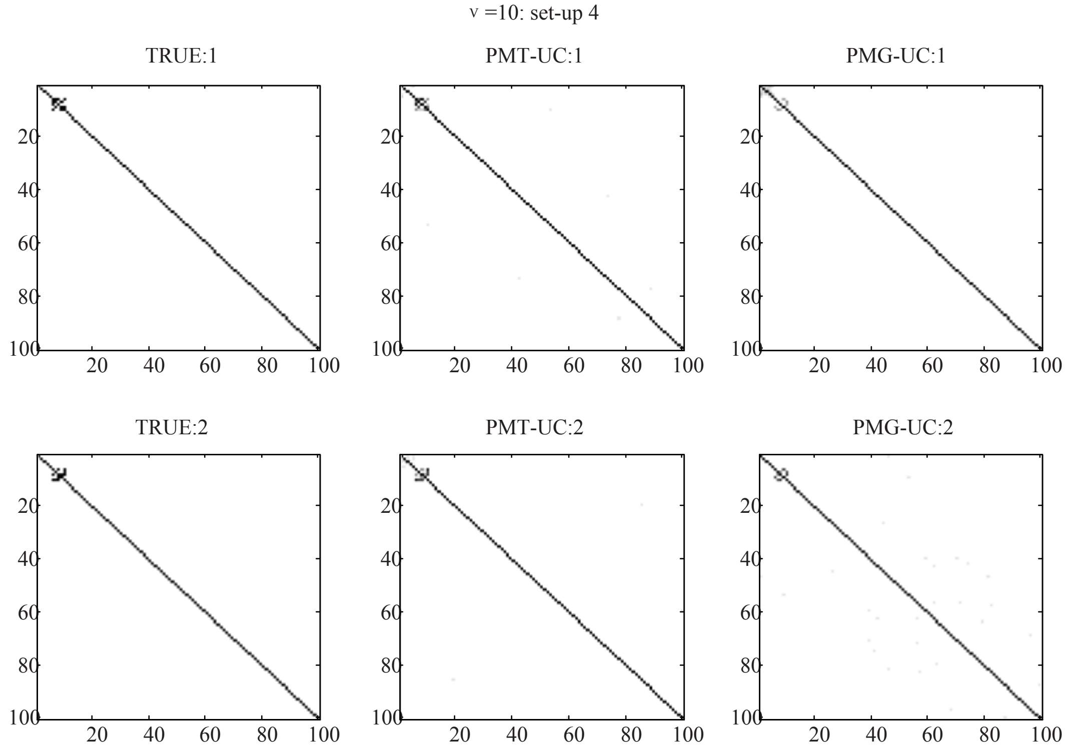

Fig. S8. Network reconstruction for simulated dataset in set-up 4 when $\nu = 10$. TRUE:1 and TRUE:2 are the original scale matrices $\boldsymbol{W}_1$ and $\boldsymbol{W}_2$ for the first and second clusters, respectively. PMT-UC:1 and PMT-UC:2 are the estimation of the scale matrices using PMT-UC. PMG-UC:1 and PMG-UC:2 are the estimation of the covariance matrices using PMG-UC.

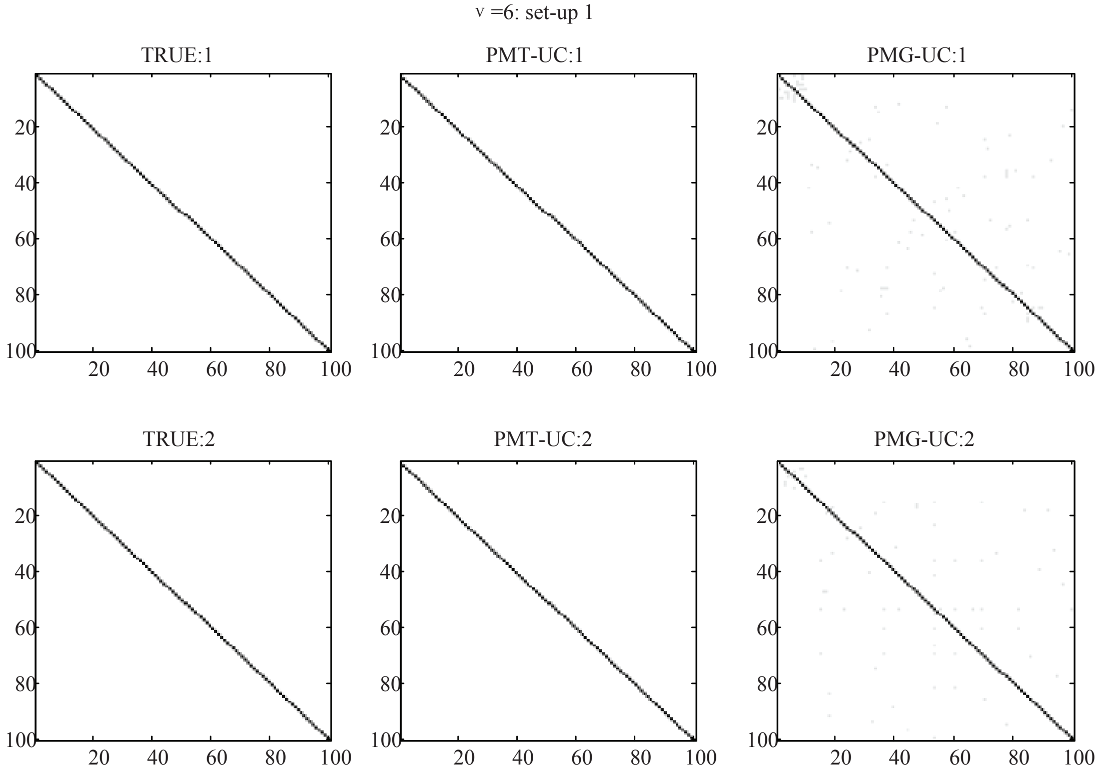

Fig. S9. Network reconstruction for simulated dataset in set-up 1 when $\nu = 6$. TRUE:1 and TRUE:2 are the original scale matrices $\boldsymbol{W}_1$ and $\boldsymbol{W}_2$ for the first and second clusters, respectively. PMT-UC:1 and PMT-UC:2 are the estimation of the scale matrices using PMT-UC. PMG-UC:1 and PMG-UC:2 are the estimation of the covariance matrices using PMG-UC.

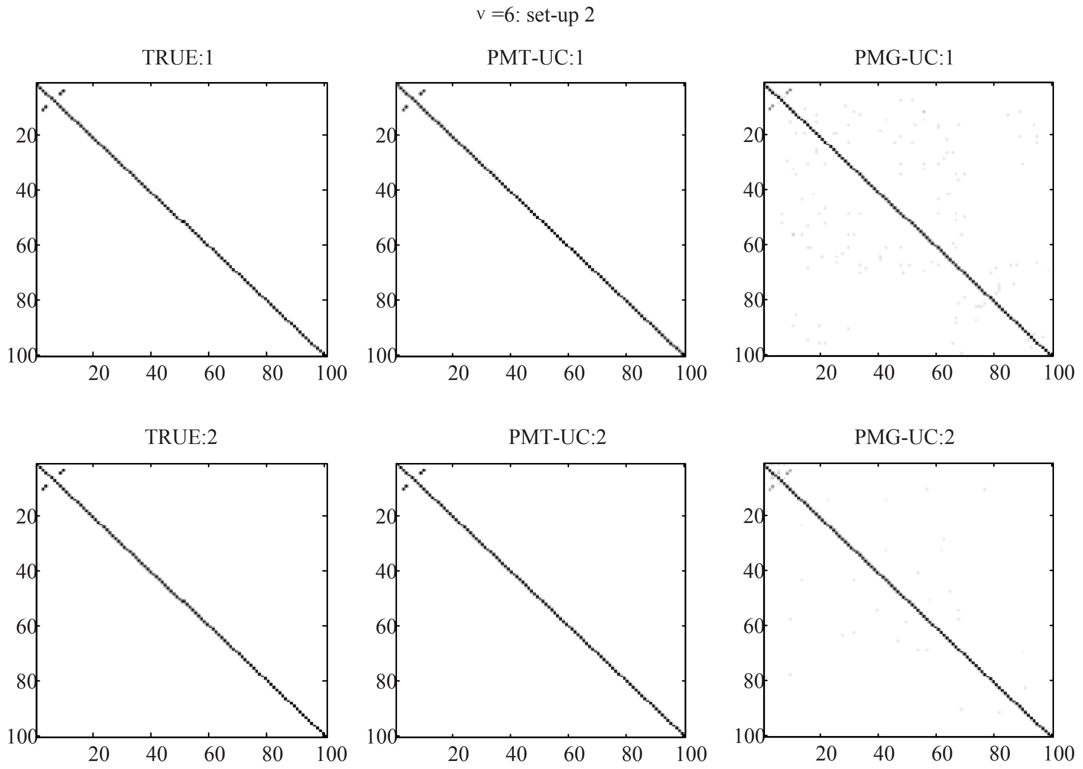

Fig. S10. Network reconstruction for simulated dataset in set-up 2 when $\nu = 6$. TRUE:1 and TRUE:2 are the original scale matrices $\boldsymbol{W}_1$ and $\boldsymbol{W}_2$ for the first and second clusters, respectively. PMT-UC:1 and PMT-UC:2 are the estimation of the scale matrices using PMT-UC. PMG-UC:1 and PMG-UC:2 are the estimation of the covariance matrices using PMG-UC.

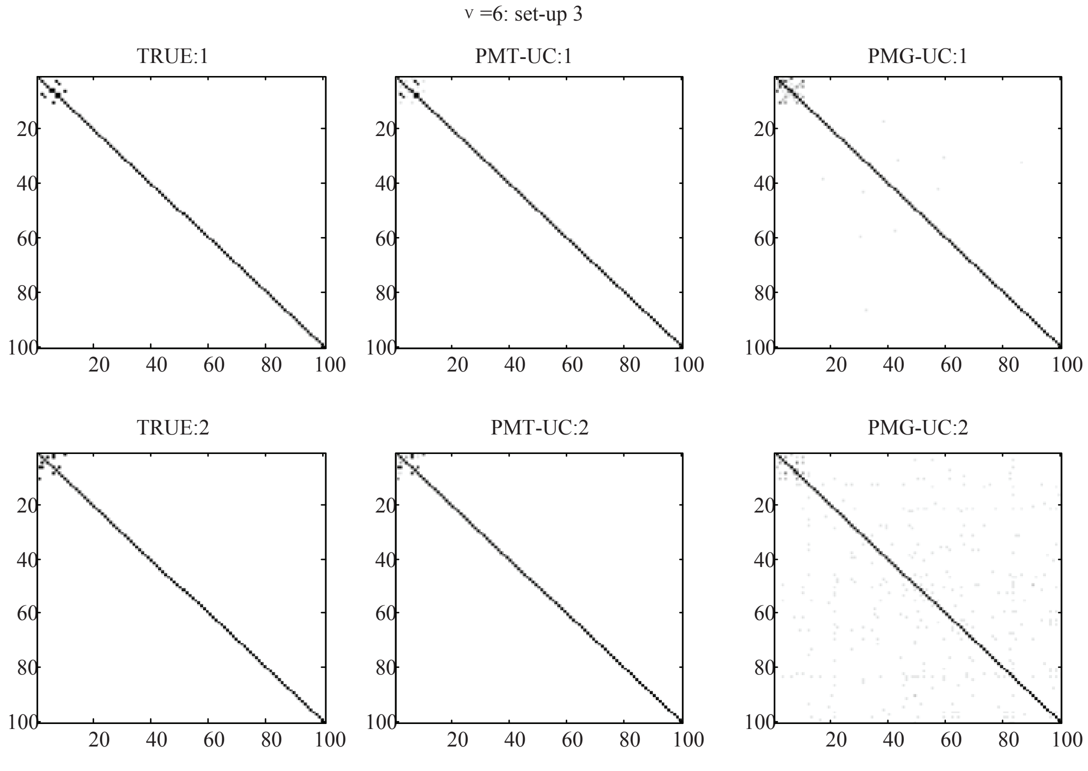

Fig. S11. Network reconstruction for simulated dataset in set-up 3 when $\nu = 6$. TRUE:1 and TRUE:2 are the original scale matrices $\boldsymbol{W}_1$ and $\boldsymbol{W}_2$ for the first and second clusters, respectively. PMT-UC:1 and PMT-UC:2 are the estimation of the scale matrices using PMT-UC. PMG-UC:1 and PMG-UC:2 are the estimation of the covariance matrices using PMG-UC.

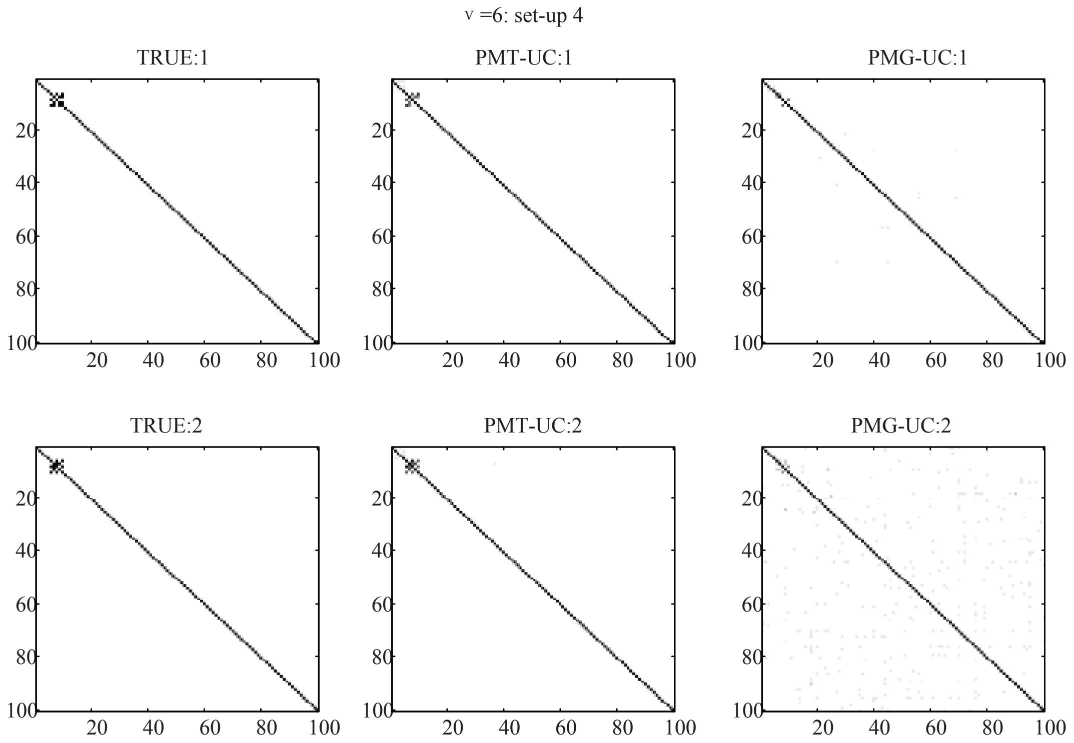

Fig. S12. Network reconstruction for simulated dataset in set-up 4 when $\nu = 6$. TRUE:1 and TRUE:2 are the original scale matrices $\boldsymbol{W}_1$ and $\boldsymbol{W}_2$ for the first and second clusters, respectively. PMT-UC:1 and PMT-UC:2 are the estimation of the scale matrices using PMT-UC. PMG-UC:1 and PMG-UC:2 are the estimation of the covariance matrices using PMG-UC.
