# Supplementary material for: Cancer Subtype Discovery and Biomarker Identification via a New Robust Network Clustering Algorithm
Source: PLoS One. 2013 Jun 17;8(6):e66256. doi: 10.1371/journal.pone.0066256 (PMC3684607; doi:10.1371/journal.pone.0066256)
Supplement: Text S5 — The detailed information of genes in the subnetworks corresponding to B-cell ALL and AML for leukemia dataset. (PDF) [file pone.0066256.s005.pdf]

The detailed information of genes in the subnetworks corresponding to B-cell ALL and AML for leukemia dataset

TABLE S1
THE DETAILED INFORMATION OF GENES IN THE SUBNETWORKS CORRESPONDING TO B-CELL ALL FOR LEUKEMIA DATASET.

Subnetwork	Affymetrix ID	Gene Symbol	mean-based discriminative gene
ALL-B-1	M13560_s_at	CD74	Y
	M33600_f_at	HLA-DRB1	Y
	HG3576-HT3779_f_at	HLA-DRB5	Y
	X17093_at	HLA-F	Y
	M57466_s_at	HLA-DPB1	Y
	X03100_cds2_at	HLA-DPA1	Y
	M34996_s_at	HLA-DQA1	N
	HG688-HT688_f_at	HLA-DRB1	Y
	X62744_at	HLA-DMA	N
ALL-B-2	M63438_s_at	IGKC	N
	M87789_s_at	IGHG3	N
	M34516_at	IGLL1	Y
	M25280_at	SELL	N
	M34516_r_at	IGLC3	N
	X57809_s_at	IGLC3	N
ALL-B-3	D32129_f_at	HLA-A	N
	M94880_f_at	HLA-A	N
	HG658-HT658_f_at	HLA-C	N
	HG2917-HT3061_f_at	HLA-E	Y
	HG2915-HT3059_f_at	HLA-E	Y
	HG3597-HT3800_f_at	HLA-B	Y
ALL-B-4	U01317_cds4_at	HBB	Y
	Z83821_cds2_at	ALAS2	N
	X77737_at	SLC4A1	Y
	U57341_at	NEFL	Y
	X64364_at	BSG	Y
	D26308_at	BLVRB	Y
ALL-B-5	S82297_at	B2M	N
	X60489_at	EEF1B2	N
	X52966_at	RPL35A	N
	X80909_at	NACA	N
	X16560_at	COX7C	N
	U90915_at	COX4I1	N
ALL-B-6	M26311_s_at	S100A9	Y
	M26602_at	DEFA1	Y
	M21005_at	S100A8	Y
ALL-B-7	X68277_at	DUSP1	N
	M62831_at	ETR101	N
	V01512_rna1_at	FOS	N
ALL-B-8	M25079_s_at	HBB	N
	Z84721_cds2_at	HBA2	N

TABLE S2
THE DETAILED INFORMATION OF GENES IN THE SUBNETWORKS CORRESPONDING TO AML FOR LEUKEMIA DATASET.

Subnetwork	Affymetrix ID	Gene Symbol	mean-based discriminative gene
AML-1	M63438_s_at	IGKC	N
	M87789_s_at	IGHG3	N
	M34516_at	IGLL1	Y
	M34516_r_at	IGLC3	N
AML-2	M92843_s_at	ZFP36	N
	X51345_at	JUNB	N
	L49169_at	FOSB	N
	U20734_s_at	JUNB	N
AML-3	J03909_at	IFI30	Y
	M33195_at	FCER1G	Y
	HG417-HT417_s_at	CTSB	Y
AML-4	Y00787_s_at	IL8	Y
	M28130_rna1_s_at	IL8	Y
	M57731_s_at	CXCL2	Y
AML-5	U01317_cds4_at	HBB	Y
	X77737_at	SLC4A1	Y
	U05255_s_at	GYPB	Y
AML-6	M23178_s_at	CCL3	Y
	J04130_s_at	CCL4	Y
	M72885_rna1_s_at	G0S2	Y
AML-7	M26311_s_at	S100A9	Y
	M21005_at	S100A8	Y
