# Supplementary material for: Cancer Subtype Discovery and Biomarker Identification via a New Robust Network Clustering Algorithm
Source: PLoS One. 2013 Jun 17;8(6):e66256. doi: 10.1371/journal.pone.0066256 (PMC3684607; doi:10.1371/journal.pone.0066256)
Supplement: Text S6 — The detailed information of all the leukemia-related biomarkers selected by PMT-UC. (PDF) [file pone.0066256.s006.pdf]

The detailed information of all the leukemia-related biomarkers selected by PMT-UC

TABLE S3: The detailed information of all the leukemia-related biomarkers selected by PMT-UC.

No.	Affymetrix ID	Gene Symbol	mean-based discriminative gene
1	M25079_s_at	HBB	N
2	Z84721_cds2_at	HBA2	N
3	M26311_s_at	S100A9	Y
4	M27891_at	CST3	Y
5	X00274_at	HLA-DRA	Y
6	M13560_s_at	CD74	Y
7	M63438_s_at	IGKC	N
8	Y00787_s_at	IL8	Y
9	HG1428-HT1428_s_at	HBB	N
10	M19507_at	MPO	Y
11	X82240_rna1_at	TCL1A	Y
12	L19779_at	HIST2H2AA	Y
13	M96326_rna1_at	AZU1	Y
14	M91036_rna1_at	HBG2	N
15	X57351_s_at	IFITM2	N
16	X56681_s_at	JUND	N
17	X00437_s_at	trbC1	Y
18	M69043_at	NFKBIA	Y
19	M11147_at	FTL	Y
20	Y00433_at	GPX1	Y
21	M33600_f_at	HLA-DRB1	Y
22	M26602_at	DEFA1	Y
23	AFFX-HUMRGE/M10098_5_at	SRP68	N
24	D64142_at	H1FX	N
25	M87789_s_at	IGHG3	N
26	Z19554_s_at	VIM	N
27	J04456_at	LGALS1	N
28	V00599_s_at	C21orf56	N
29	Z48950_at	H3F3B	N
30	M21186_at	CYBA	N
31	D32129_f_at	HLA-A	N
32	AFFX-M27830_5_at		Y
33	D21261_at	TAGLN2	Y
34	L06797_s_at	CXCR4	Y
35	M12886_at	IL23A	Y
36	J03077_s_at	PSAP	Y
37	HG3576-HT3779_f_at	HLA-DRB5	Y
38	U01317_cds4_at	HBB	Y
39	Z48501_s_at	PABPC1	N
40	X58529_at	IGHM	Y
41	X69654_at	RPS26	N
42	M21005_at	S100A8	Y
43	M94880_f_at	HLA-A	N
44	L20941_at	FTH1	Y
45	AFFX-M27830_M_at	NAN	N
46	M13792_at	ADA	Y
47	X68277_at	DUSP1	N
48	M14328_s_at	ENO1	N
49	M27783_s_at	ELANE	Y
50	V00594_s_at	MT2A	N
51	X14008_rna1_f_at	LYZ	Y
52	M19045_f_at	LYZ	Y
53	X78992_at	ZFP36L2	N

Continued on next page

TABLE S3 – continued from previous page

No.	Affymetrix ID	Gene Symbol	mean-based discriminative gene
54	J04164_at	IFITM1	N
55	HG658-HT658_f_at	HLA-C	N
56	S73591_at	TXNIP	N
57	M20203_s_at	ELANE	Y
58	M14483_rna1_s_at	PTMA	Y
59	M28130_rna1_s_at	IL8	Y
60	J03801_f_at	LYZ	Y
61	AFFX-HUMGAPDH/M33197_5_at	GAPD	Y
62	L26247_at	EIF1	N
63	AFFX-HUMGAPDH/M33197_M_at	GAPD	N
64	M34516_at	IGLL1	Y
65	AFFX-HSAC07/X00351_5_at	ACTB	Y
66	Z23090_at	HSPB1	N
67	J04990_at	CTSG	Y
68	M16279_at	CD99	N
69	M21119_s_at	LYZ	Y
70	X76223_s_at	MAL	Y
71	U51240_at	LAPTM5	Y
72	M62831_at	ETR101	N
73	HG2917-HT3061_f_at	HLA-E	Y
74	HG2915-HT3059_f_at	HLA-E	Y
75	D63874_at	HMGB1	Y
76	M33680_at	CD81	Y
77	M84526_at	DF	Y
78	AFFX-HUMRGE/M10098_M_at	GPR34	Y
79	D78361_at	OAZ1	N
80	M23613_at	NPM1	N
81	S71043_rna1_s_at	LOC100126583	Y
82	X15183_at	HSPCA	N
83	S82297_at	B2M	N
84	X17042_at	SRGN	Y
85	M11722_at	DNTT	Y
86	S54005_s_at	TMSB10	N
87	J03592_at	SLC25A6	N
88	AFFX-HUMRGE/M10098_3_at	SRP68	Y
89	U14971_at	RPS9	N
90	X12447_at	ALDOA	N
91	U05259_rna1_at	CD79A	Y
92	U52101_at	EMP3	N
93	X17093_at	HLA-F	Y
94	X67951_at	PRDX1	Y
95	X00351_f_at	ACTB	Y
96	HG2815-HT2931_at	MYL6	N
97	X60489_at	EEF1B2	N
98	X55668_at	PRTN3	Y
99	M19311_s_at	CALM2	N
100	Z83821_cds2_at	ALAS2	N
101	V01512_rna1_at	FOS	N
102	M92843_s_at	ZFP36	N
103	U23852_s_at	LCK	Y
104	U43901_rna1_s_at	RPSA	Y
105	D88270_at	VPREB1	Y
106	M63138_at	CTSD	Y
107	V00563_at	IGHM	Y
108	U14603_at	PTP4A2	Y
109	M21142_cds2_s_at	GNAS	Y
110	J03191_at	PFN1	N
111	X65965_s_at	SOD2	Y
112	U58682_at	RPS28	N
113	M21305_at		Y
114	HG3597-HT3800_f_at	HLA-B	Y
115	D49824_s_at	HLA-B	N
116	X03934_at	CD3D	Y
117	X51345_at	JUNB	N
118	X03689_s_at	EEF1A1	Y
119	X78136_at	PCBP2	N
120	U00947_s_at	HNRPA1	N

Continued on next page

TABLE S3 – continued from previous page

No.	Affymetrix ID	Gene Symbol	mean-based discriminative gene
121	M92287_at	CCND3	Y
122	J03909_at	IFI30	Y
123	X77737_at	SLC4A1	Y
124	X03068_f_at	HLA-DQB1	Y
125	D00749_s_at	CD7	Y
126	M69066_at	MSN	N
127	X12671_rna1_at	HNRPA1	N
128	M26880_at	UBC	N
129	X62320_at	GRN	Y
130	U49835_s_at	CHI3L2	Y
131	HG311-HT311_at	RPL24	N
132	U15008_at	SNRPD2	N
133	M86400_at	YWHAZ	N
134	X52966_at	RPL35A	N
135	HG2788-HT2896_at	S100A6	Y
136	J04988_at	HSPCB	N
137	U89922_s_at	LTB	Y
138	M80563_at	S100A4	Y
139	X80909_at	NACA	N
140	AFFX-HSAC07/X00351_M_at	ACTB	Y
141	X04500_at	IL1B	Y
142	X02152_at	LDHA	N
143	U05255_s_at	GYPB	Y
144	L11672_at	ZNF91	N
145	M57466_s_at	HLA-DPB1	Y
146	L49169_at	FOSB	N
147	X59417_at	PSMA6	Y
148	M25280_at	SELL	N
149	M34516_r_at	IGLC3	N
150	Z49148_s_at	RPL29	N
151	X03100_cds2_at	HLA-DPA1	Y
152	X57809_s_at	IGLC3	N
153	M59807_at	IL32	Y
154	S77356_at	ATP5O	N
155	D13748_at	EIF4A1	N
156	S53911_at	CD34	N
157	U29607_at	METAP2	N
158	U50743_at	FXYD2	Y
159	M21624_at	TRAC	Y
160	M60750_f_at	HIST1H2BG	Y
161	M57710_at	LGALS3	Y
162	D14710_at	ATP5A1	N
163	U93205_at	CLIC1	N
164	U46751_at	SQSTM1	Y
165	HG1980-HT2023_at	TUBB2	N
166	HG662-HT662_at	RPL22	N
167	Z15115_at	TOP2B	Y
168	M31627_at	XBP1	Y
169	M13934_cds2_at	RPS14	N
170	D88422_at	CSTA	Y
171	M21388_r_at	IGHG3	N
172	M91438_at	SPINK2	Y
173	M54915_s_at	PIM1	Y
174	M24485_s_at	GSTP1	Y
175	L19686_rna1_at	MIF	N
176	U06155_s_at	RPL23AP7	Y
177	X70683_at	SOX4	N
178	X13794_rna1_at	LDHB	N
179	D00017_at	ANXA2	N
180	V00594_at	MT2A	Y
181	U22376_cds2_s_at	MYB	Y
182	X04085_rna1_at	CAT	Y
183	U02493_at	NONO	Y
184	L17131_rna1_at	HMGA1	N
185	M23178_s_at	CCL3	Y
186	D30655_at	EIF4A2	Y
187	J04130_s_at	CCL4	Y

Continued on next page

TABLE S3 – continued from previous page

No.	Affymetrix ID	Gene Symbol	mean-based discriminative gene
188	HG1862-HT1897_at	CALM1	Y
189	AF000424_s_at	LST1	Y
190	D50310_at	CCNI	Y
191	X64072_s_at	ITGB2	Y
192	J02683_s_at	SLC25A5	N
193	U20734_s_at	JUNB	N
194	J04823_rna1_at	COX8	Y
195	X01677_f_at	GAPD	N
196	J03779_at	MME	Y
197	L33930_s_at	CD24	Y
198	HG1612-HT1612_at	MARCKSL1	Y
199	HG2279-HT2375_at	TPI1	N
200	X13546_rna1_at	HMGN2	N
201	L09604_at	PLP2	N
202	U68105_s_at	PABPC1	N
203	D21260_at	CLTC	Y
204	L21954_at	BZRP	Y
205	HG3514-HT3708_at	TPM3	N
206	L25080_at	ARHA	N
207	D83920_at	FCN1	Y
208	U60644_at	PLD3	Y
209	X65977_at	DEFA4	Y
210	U70439_s_at	ANP32B	N
